# Complete xylan utilization pathway and regulation mechanisms involved in marine algae degradation by cosmopolitan marine and human gut microbiota

**DOI:** 10.1093/ismejo/wraf085

**Published:** 2025-05-22

**Authors:** Hai-Ning Sun, Xiu-Lan Chen, Yan Wang, Yan-Ping Zhu, Zhao-Jie Teng, Hai-Yan Cao, Ting-Ting Xu, Yin Chen, Yu-Zhong Zhang, Fang Zhao

**Affiliations:** State Key Laboratory of Microbial Technology, Shandong University, Qingdao 266237, Shandong Province, China; State Key Laboratory of Microbial Technology, Shandong University, Qingdao 266237, Shandong Province, China; State Key Laboratory of Microbial Technology, Shandong University, Qingdao 266237, Shandong Province, China; State Key Laboratory of Microbial Technology, Shandong University, Qingdao 266237, Shandong Province, China; State Key Laboratory of Microbial Technology, Shandong University, Qingdao 266237, Shandong Province, China; State Key Laboratory of Microbial Technology, Shandong University, Qingdao 266237, Shandong Province, China; MOE Key Laboratory of Evolution and Marine Biodiversity, Frontiers Science Center for Deep Ocean Multispheres and Earth System & College of Marine Life Sciences, Ocean University of China, Qingdao 266003, Shandong Province, China; State Key Laboratory of Microbial Technology, Shandong University, Qingdao 266237, Shandong Province, China; MOE Key Laboratory of Evolution and Marine Biodiversity, Frontiers Science Center for Deep Ocean Multispheres and Earth System & College of Marine Life Sciences, Ocean University of China, Qingdao 266003, Shandong Province, China; School of Life Sciences, University of Warwick, Coventry CV4 7AL, West Midlands, United Kingdom; MOE Key Laboratory of Evolution and Marine Biodiversity, Frontiers Science Center for Deep Ocean Multispheres and Earth System & College of Marine Life Sciences, Ocean University of China, Qingdao 266003, Shandong Province, China; Laboratory for Marine Biology and Biotechnology, Qingdao Marine Science and Technology Center & Laoshan Laboratory, Qingdao 266237, Shandong Province, China; Marine Biotechnology Research Center, State Key Laboratory of Microbial Technology, Shandong University, Qingdao 266237, Shandong Province, China; State Key Laboratory of Microbial Technology, Shandong University, Qingdao 266237, Shandong Province, China

**Keywords:** β-1,3-xylan, catabolism pathway, marine algae, marine bacteria, human gut microbiota

## Abstract

β-1,3-xylan, typically found in marine algae as a major cell wall polysaccharide, represents an overlooked pool of organic carbon in global oceans. Whilst our understanding of microbial catabolism of xylans has improved significantly, particularly from biotransformations of terrestrial plant biomass that are typically composed of β-1,4-xylans, knowledge on how microbes utilize β-1,3-xylan remains limited. Here, we describe the discovery of a complete pathway for β-1,3-xylan catabolism and its regulation in a marine bacterium, *Vibrio* sp. EA2. The pathway starts with the extracellular decomposition of β-1,3-xylan by two β-1,3-xylanases into β-1,3-xylooligomers, which are mainly internalized by an ATP-binding cassette transporter. The substrate binding protein of this transporter has an L-shaped substrate binding pocket to preferentially bind β-1,3-xylooligomers. Subsequently, two intracellular β-1,3-xylosidases degrade β-1,3-xylooligomers into fermentable xylose. The pathway is activated by a unique regulator with xylose being the effector. This β-1,3-xylan catabolic pathway differs from that of β-1,4-xylan catabolism in enzymes, transporters, and regulators. Bioinformatic analysis suggests that the β-1,3-xylan catabolism pathway is not only prevalent in diverse marine bacteria and cosmopolitan human gut microbiota, such as *Bacteroides*, but also likely transferred horizontally from algae-degrading marine bacteria to the human gut.

## Introduction

Marine algae generate ~105 Gt organic carbon annually, primarily deposited as polysaccharides [1]. These algal polysaccharides are mainly used as carbon sources by marine heterotrophic bacteria [2, 3]. The degradation of algal polysaccharides by marine bacteria represents one of the largest biotransformations on Earth, making it a key component in the global carbon cycle [4, 5].

β-1,3-xylan, a homoxylan composed of β-1,3-linked D-xylose, is an algal polysaccharide absent in terrestrial plants [6–9]. It is a main component of the cell walls of many green (*Caulerpa*, *Dichotomosiphon*, *Halimeda*, *Penicillus*, *Udotea* spp., and others) and red algae (*Porphyra*, *Bangia* spp., and others). The cell walls of these algae are devoid of cellulose but instead reinforced by crystalline microfibrils of β-1,3-xylan [8]. β-1,3-xylan-containing algae are globally distributed, especially in coastal oceans (http://algaebase.org), among which *Caulerpa* and *Porphyra* are noteworthy. Many *Caulerpa* species are strong invaders with *Caulerpa taxifolia* being the most notorious and threatening one, once covering 30 000 hectares of coastal sea floor in the Mediterranean [10–12]. In addition, many *Caulerpa* and *Porphyra* species are widely cultivated for human consumption, such as *Caulerpa lentillifera* and *Porphyra yezoensis*. Therefore, as a main cell wall component of these algae, β-1,3-xylan constitutes a huge biomass and a carbon source for marine heterotrophic bacteria [13]. Nevertheless, the mechanism of β-1,3-xylan catabolism by marine bacteria remains poorly studied, although some β-1,3-xylanases [14–22] and β-1,3-xylosidases [23, 24] have been reported to degrade β-1,3-xylan and β-1,3-xylooligosaccharides (β-1,3-XOs), respectively.

**Figure 1:**
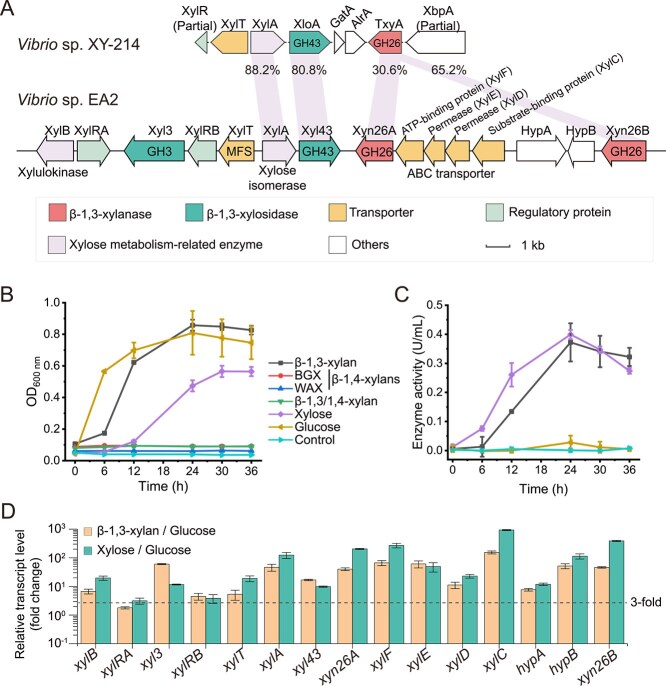
Identification of the β-1,3-xylan utilization locus in strain EA2. (A) Synteny of β-1,3-xylan utilization loci in *vibrio* sp. XY-214 and *vibrio* sp. EA2. (B) Growth curves of strain EA2 on different classes of xylans (β-1,3-xylan, BGX, WAX, and β-1,3/1,4-xylan) and the control substrates (xylose and glucose) with a final concentration of 0.2% (w/v). The culture without a carbon source was treated as the control. (C) Extracellular β-1,3-xylanase activities during the cultivation of strain EA2 on β-1,3-xylan, xylose, and glucose. β-1,3-xylanase activities were determined at 30°C in PBS (20 mM, pH 7.0). (D) RT-qPCR analysis of the transcriptional levels of genes in the β-1,3-xylan utilization locus of strain EA2 in response to β-1,3-xylan and xylose, relative to glucose. The *recA* gene was used as an internal reference. The data shown in the graphs are from triplicate experiments (mean ± SD).

*Vibrio* species are heterotrophic bacteria ubiquitous in marine environments and play an important ecological role in the marine carbon cycle as potent polysaccharide degraders [25, 26]. *Vibrio* species are dominant members catabolizing β-1,3-xylan [13]. Several *Vibrio* strains have been reported to secrete β-1,3-xylanases and utilize β-1,3-xylan [13, 15, 17]. In *Vibrio* sp. XY-214, a gene cluster involved in β-1,3-xylan catabolism has been reported (Fig. 1A) [27], and the enzymes encoded by genes *txyA* (a β-1,3-xylanase) [15], *xloA* (a β-1,3-xylosidase) [23], and *xylA* (a xylose isomerase) [27], have been biochemically characterized. However, the complete pathway for β-1,3-xylan catabolism of *Vibrio* remains elusive.

*Vibrio* sp. strain EA2 is a β-1,3-xylan-utilizing bacterium isolated from rotten *C. lentillifera* [13]. Here, we describe the discovery and the integrative characterization of a complete pathway for β-1,3-xylan catabolism and its regulation in strain EA2, which was shown to be different from bacterial catabolism of β-1,4-xylan and other polysaccharides. Furthermore, we systematically examined bacterial genomes and metagenomes, and revealed the widespread distribution of β-1,3-xylanolytic systems among marine bacteria, with evidence of transfer into human gut microbiota.

## Materials and methods

### Materials and bacterial strains

β-1,3-xylan, β-1,3-xylobiose (β-1,3X2), β-1,3-xylotriose (β-1,3X3), and β-1,3-xylotetraose (β-1,3X4) were prepared as previously described [13]. Beechwood glucuronoxylan (BGX), wheat arabinoxylan (WAX), β-1,4-xylobiose (β-1,4X2), and β-1,4-xylotriose (β-1,4X3) were purchased from Megazyme (Ireland). β-1,3/1,4-mixed-linkage xylan was purchased from Elicityl (France). Strain EA2 was previously isolated from a rotten *C. lentillifera* sample collected from Hainan, China [13].

### Growth experiments and extracellular β-1,3-xylanase activity assays

Growth of strain EA2 and extracellular β-1,3-xylanase activity assays were performed with the methods previously described [13]. Briefly, after cultivation in the marine broth (MB) medium at 30°C to an OD_600_ of 1.0, bacterial cells were washed twice and resuspended in artificial seawater. Then, cells were inoculated into the medium with one of the following carbon sources as the sole carbon source: β-1,3-xylan, BGX, WAX, β-1,3/1,4-xylan, β-1,3X2, β-1,3X3, xylose, and glucose. Detailed protocols for growth experiments are described in Supplementary Materials and Methods. Extracellular β-1,3-xylanase activities in the culture supernatants were determined at 30°C in the phosphate-buffered saline (PBS; 20 mM, pH 7.0) using the dinitrosalicylic acid (DNS) method [28].

### Real-time quantitative PCR

Strain EA2 was cultivated on β-1,3-xylan, xylose, and glucose, separately, to the exponential phase. Cells were collected for the extraction of total RNA and real-time quantitative PCR (RT-qPCR). Detailed protocols for RNA extraction and RT-qPCR are described in Supplementary Materials and Methods. The primers used are listed in Table S1.

### Genetic manipulation

Gene knockouts were performed by homologous recombination according to a previously published method [29]. Detailed protocols for gene knockouts and gene complementation are described in Supplementary Materials and Methods. The primers used are listed in Table S1 and the strains and plasmids are in Table S2.

### Gene cloning, site-directed mutagenesis, protein production, and purification

Genes were cloned into the pET-22b vector containing a His tag and overexpressed in *Escherichia coli* BL21(DE3). Proteins were purified by nickel affinity chromatography. Site-directed mutations were introduced using the QuikChange II mutagenesis kit (Agilent, USA). Detailed protocols are described in Supplementary Materials and Methods.

### Enzyme assays and characterization

Unless otherwise noted, enzyme assays were performed in PBS (20 mM) under their respective optimum pHs and temperatures. The xylanase activity was determined with xylans (β-1,3-xylan, BGX, WAX, or β-1,3/1,4-xylan) as the substrates using the DNS method [28]. The xylosidase activity was determined with p-nitrophenyl-β-D-xylopyranoside or XOs (β-1,3X2, β-1,3X3, β-1,4X2, or β-1,4X3) as the substrate [30]. The activity of xylose isomerase was determined with xylose as the substrate by the cysteine-carbazole method [31]. The activity of xylulokinase was determined with xylulose as the substrate in a multienzyme reaction system [32]. Product analysis of β-1,3-xylanases was performed using size-exclusion chromatography on a Superdex 30 Increase 10/300 GL column (GE Healthcare, USA) [13]. Products from xylose by the catalysis of XylA and XylB were analyzed with high-resolution Q-TOF mass spectrometry (Q-TOF-MS) (Bruker Impact HD, Germany). Detailed protocols are described in Supplementary Materials and Methods.

### Isothermal titration calorimetry

Isothermal titration calorimetry (ITC) measurements were performed at 25°C using a MicroCal PEAQ-ITC (Malvern, United Kingdom) in 10 mM Tris–HCl (pH 8.0) containing 100 mM NaCl. Data were analyzed with the Microcal PEAQ-ITC analysis software. Detailed protocols are described in Supplementary Materials and Methods.

### Crystallization, data collection, and structure determination

Crystal structures of XylC in complex with β-1,3X2, β-1,3X3, and β-1,4X2 were determined by molecular replacement using the CCP4 program Phaser [33]. The structure of XylC predicted by Alpha-Fold2 [34] was used as the search model. Detailed protocols are described in Supplementary Materials and Methods.

### Electrophoretic mobility shift assay

Electrophoretic mobility shift assay (EMSA) was conducted according to a previously published method [35]. DNA probes (50 bp) containing the predicted XylRA binding sites were labeled with 5'-Biotin. The sequences of DNA probes are listed in Table S1. Detailed protocols are described in Supplementary Materials and Methods.

### Bioinformatics

XylRA homologs were extracted from NCBI clustered nr database with a cut-off value of *E* value <1e^−50^, identity >50%, and coverage >75%. All available bacterial genomes in Integrated Microbial Genome database (IMG) database [13] were probed for the homologs of Xyn26A and Xyn26B with a cut-off value of *E* value <1e^−50^ and identity >35%. The cut-off values were scrutinized by multiple sequence alignment, structural analysis, and/or biochemical characterization. For metagenomic analysis, a total of 3521 datasets of marine and human gut origins were used. Horizontal gene transfer (HGT) events were predicted via RANGER-DTL 2.0, a software package which takes as input a gene tree and a species tree and reconciles the two to infer gene evolutionary events [36]. Detailed protocols are described in Supplementary Materials and Methods.

## Results and discussion

### Identification of a β-1,3-xylan utilization locus in strain EA2

There are structurally diverse xylans in the ocean, including β-1,3-xylan, mixed-linkage β-1,3/1,4-xylan, and β-1,4-xylan [37, 38]. Growth assays showed that strain EA2 could utilize β-1,3-xylan and xylose as the sole carbon source for growth, but not β-1,3/1,4-xylan or β-1,4-xylans (BGX and WAX) (Fig. 1B), indicating that strain EA2 is a catabolic specialist to β-1,3-xylan. The extracellular β-1,3-xylanase activity of strain EA2 was induced upon growth on both β-1,3-xylan and xylose (Fig. 1C).

Genomic analysis of strain EA2 (NCBI accession no. ASM3304249v1) revealed that 15 genes potentially involved in β-1,3-xylan catabolism are packaged in a single gene cluster (locus tag: RXX31_21090–21170) (Fig. 1A and Table S3). Based on functional annotation, this cluster encodes two β-1,3-xylanases (Xyn26A and Xyn26B of the GH26 family), two xylosidases (Xyl3 of the GH3 family and Xyl43 of the GH43 family), two transporters (XylT of the major facilitator superfamily [MFS] and XylCDEF of the ATP-binding cassette [ABC] family), two transcriptional regulators (XylRA and XylRB), one xylose isomerase (XylA), one xylulokinase (XylB), and two hypothetical proteins (HypA and HypB). RT-qPCR analysis showed that the transcriptional levels of most genes in this cluster were significantly upregulated (≥ 3-fold) in the cells cultivated with β-1,3-xylan and xylose, relative to glucose (Fig. 1D), suggesting that this gene cluster is critical for β-1,3-xylan catabolism. These results suggest that strain EA2 assembles genes required for β-1,3-xylan catabolism within a gene cluster, which was termed *xul* in this study. This is a common strategy adopted by bacteria to consume polysaccharides, known as the polysaccharide utilization loci (PULs) [39].

Only *Vibrio* sp. XY-214 has been reported to utilize β-1,3-xylan by a PUL [27]. However, the genomic data of this strain is not available, and its β-1,3-xylan utilization locus was obtained by Southern hybridization. In contrast to *xul* that contains 15 genes, the locus of *Vibrio* sp. XY-214 contains eight genes organized in the following order: *xylR*, *xylT*, *xylA*, *xloA*, *gatA*, *alrA*, *txyA*, and *xbpA*, of which *xylR* and *xbpA* are incomplete open reading frames (ORFs) (Fig. 1A) [27]. The sequence information and biochemical data of only three gene products from this locus are available in databases, including the xylose isomerase XylA [27], the β-1,3-xylosidase XloA [23], and the β-1,3-xylanase TxyA [15], all of which are homologous to the corresponding *xul*-encoded enzymes.

### Enzymatic catabolism of β-1,3-xylan by strain EA2

β-1,3-xylanases and β-1,3-xylosidases catalyze the degradation of β-1,3-xylan and β-1,3-XOs, respectively [14, 23]. Currently, 10 β-1,3-xylanases (all from GH26) [14–22] and two β-1,3-xylosidases (both from GH43) [23, 24] have been biochemically characterized. Based on functional annotation, *xul* encodes two β-1,3-xylanases (Xyn26A and Xyn26B) and two β-1,3-xylosidases (Xyl3 and Xyl43) (Fig. 1A).

**Figure 2:**
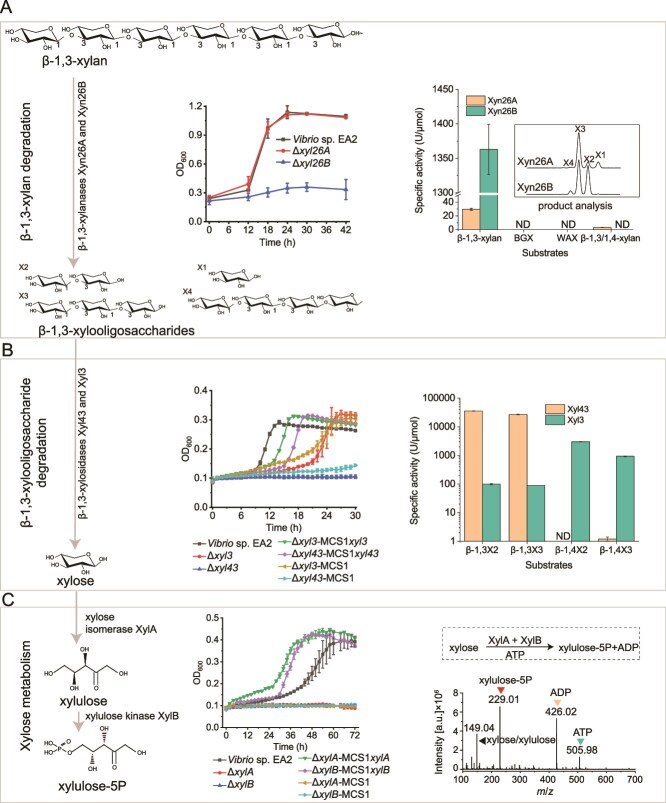
Enzymatic catabolism of β-1,3-xylan by strain EA2. (A) *In vivo* (left) and *in vitro* (right) characterization of β-1,3-xylanases Xyn26A and Xyn26B. The wild-type strain EA2 and the gene deletion mutants (Δ*xyn26A* and Δ*xyn26B*) were cultivated with 0.2% (w/v) β-1,3-xylan as the sole carbon source. To analyze enzymatic products, treatments of β-1,3-xylan by Xyn26A (105.0 μM) or Xyn26B (2.6 μM) were carried out at 30°C in PBS (20 mM, pH 7.0) for 24 h. BGX, beechwood glucuronoxylan; WAX, wheat arabinoxylan; ND, no detectable enzyme activity. (B) *In vivo* (left) and *in vitro* (right) characterization of β-1,3-xylosidases Xyl43 and Xyl3. The wild-type strain EA2, the gene deletion mutants (Δ*xyl43* and Δ*xyl3*), the complement mutants (Δ*xyl43*-MCS1*xyl43* and Δ*xyl3*-MCS1*xyl3*), and the mutants complemented with an empty plasmid (Δ*xyl43*-MCS1 and Δ*xyl3*-MCS1) were cultivated with 2 mM β-1,3X3 as the sole carbon source. (C) *In vivo* (left) and *in vitro* (right) characterization of the xylose isomerase XylA and the xylulokinase XylB. The wild-type strain EA2, the gene deletion mutants (Δ*xylA* and Δ*xylB*), the complement mutants (Δ*xylA*-MCS1*xylA* and Δ*xylB*-MCS1*xylB*), and the mutants complemented with an empty plasmid (Δ*xylA*-MCS1 and Δ*xylB*-MCS1) were cultivated with 5 mM xylose as the sole carbon source. The negative-mode Q-TOF-MS spectrum showed the products from xylose catalyzed by the recombinant XylA and XylB. The data shown in the graphs are from triplicate experiments (mean ± SD or representative data).

Prediction by SignalP 5.0 indicated that both Xyn26A and Xyn26B have a signal peptide (Table S3), suggesting that they are extracellular proteins. Xyn26A displays the highest amino acid similarity (30.6%) to the β-1,3-xylanase TxyA from *Vibrio* sp. XY-214 [15], and Xyn26B to the β-1,3-xylanase Xyl4 (95.6%) from *Vibrio* sp. AX-4 [17]. Genetic analyses showed that the *xyn26A*-deletion mutant strain Δ*xyn26A* displayed a similar growth to the wild-type (WT) strain EA2, and the *xyn26B*-deletion mutant strain Δ*xyn26B* was unable to grow on β-1,3-xylan (Fig. 2A), suggesting that *xyn26B* likely plays a major role in β-1,3-xylan utilization under this experimental condition. Unfortunately, the growth of Δ*xyn26B* on β-1,3-xylan was not restored by complementing *xyn26B* as well as its promoter region with different lengths (0.2–1.0 kb), and further attempts are needed. Recombinant Xyn26A and Xyn26B were produced in *E. coli* and purified by Ni-affinity (Fig. S1a). Xyn26A and Xyn26B both displayed the maximum β-1,3-xylanase activity at 40°C and pH 6.0 (Fig. S1b). Xyn26A had an 8.9-fold higher activity on β-1,3-xylan (29.5 U/μmol) over β-1,3/1,4-xylan (3.3 U/μmol), and no activity on β-1,4-xylan BGX or WAX (Fig. 2A). Xyn26B degraded only β-1,3-xylan with an activity of 1363.0 U/μmol, 46.2-fold higher than that of Xyn26A. These results demonstrate that both Xyn26A and Xyn26B are robust β-1,3-xylanases. Product analysis showed that both Xyn26A and Xyn26B are endolytic enzymes, with β-1,3X3 and a mixture of β-1,3X2 and β-1,3X3, respectively, as the main products when degrading β-1,3-xylan (Fig. 2A). Consistently, when β-1,3-xylan was degraded by the extracellular enzymes secreted by strain EA2, the products are mainly β-1,3X2 and β-1,3X3 [13].

Neither Xyl43 nor Xyl3 have a signal peptide, suggesting that they are cytoplasmic proteins. Xyl43 shares the highest amino acid similarity (80.8%) to the β-1,3-xylosidase XloA from *Vibrio* sp. XY-214 [15], and Xyl3 to the β-xylosidase P24_GH3 (35.3%) from *Formosa agariphila* that degrades ulvan oligosaccharides [40]. The deletion of either *xyl43* (Δ*xyl43*) or *xyl3* (Δ*xyl3*) affected the growth of strain EA2 on β-1,3X3, and *xyl43* deletion had a greater effect (Fig. 2B). Growths of Δ*xyl43* and Δ*xyl3* were partially restored by complementing the corresponding gene. These results suggest that *xyl43* and *xyl3* are involved in β-1,3-XOs utilization. Recombinant Xyl43 displayed the maximum activity at 30°C and pH 7.0, and Xyl3 at 40°C and pH 7.0 (Fig. S1). Xyl43 efficiently hydrolyzed β-1,3X2 (35586.0 U/μmol) and β-1,3X3 (26859.2 U/μmol), with negligible activity on β-1,4X2 or β-1,4X3 (Fig. 2B), indicating that it is a strict β-1,3-xylosidase. In contrast, Xyl3 had noticeable activity on all tested XOs including β-1,3X2, β-1,3X3, β-1,4X2 and β-1,4X3, with higher activities on β-1,4-XOs (932.3–3003.7 U/μmol) than β-1,3-XOs (89.0–99.2 U/μmol) (Fig. 2B), suggesting that it has a broad substrate spectrum.

XylA and XylB share the highest amino acid similarity, respectively, to the xylose isomerase XylA from *Vibrio* sp. XY-214 (88.2%) [27] and the xylulose kinase from *E. coli* (64.9%) [41]. Genetic analyses showed that gene deletion mutant strains Δ*xylA* and Δ*xylB* both lost their ability to grow on xylose, and their growths were restored by complementing the corresponding gene (Fig. 2C), suggesting that *xyl43* and *xyl3* are essential in xylose metabolism. Biochemical data indicated that XylA is a xylose isomerase with the highest activity at 50°C and pH 7.0 (309.3 ± 10.7 U/μmol), and XylB is a xylulose kinase with the highest activity at 40°C and pH 8.0 (912.0 ± 172.6 U/μmol) (Fig. S1). Q-TOF-MS revealed that XylA and XylB could catalyze xylose to produce xylulose-5-phosphate (Fig. 2C), further validating the function of XylA and XylB. Therefore, strain EA2 metabolizes xylose by the xylose isomerase pathway, as that in other bacteria [42].

Taken together, the above results suggest that strain EA2 degrades β-1,3-xylan through an enzymatic cascade involving β-1,3-xylanases (Xyn26A and Xyn26B) and β-1,3-xylosidases (Xyl3 and Xyl43). Specifically, extracellularly β-1,3-xylan is primarily decomposed by Xyn26B into β-1,3-XOs. The generated β-1,3-XOs are completely saccharified into xylose intracellularly by Xyl3 and Xyl43. The produced xylose enters the canonical xylose isomerase pathway to be further metabolized by the xylose isomerase XylA and the xylulose kinase XylB.

### Uptake of β-1,3-xylooligosaccharides by strain EA2

**Figure 3:**
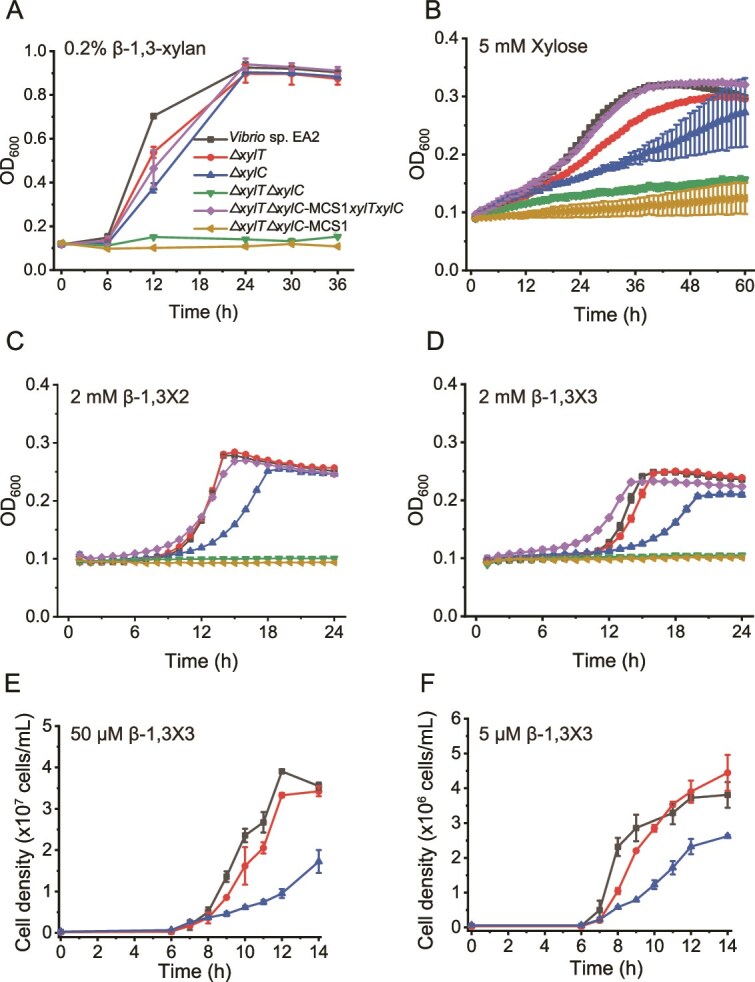
Identification of β-1,3-xylooligosaccharides transporters in strain EA2 based on genetic analysis. The wild-type strain EA2, the single/double gene deletion mutants (Δ*xylT*, Δ*xylC*, and Δ*xylT*Δ*xylC*), the complement mutant (Δ*xylT*Δ*xylC*-MCS1*xylTxylC*), and the Δ*xylT*Δ*xylC* mutant complemented with an empty plasmid (Δ*xylT*Δ*xylC*-MCS1) were cultivated with 0.2% (w/v) β-1,3-xylan (A), 5 mM xylose (B), 2 mM β-1,3X2 (C), 2 mM β-1,3X3 (D), 50 μM β-1,3X3 (E), or 5 μM β-1,3X3 (F) as the sole carbon source. The data shown in the graphs are from triplicate experiments (mean ± SD).

The above results demonstrate that strain EA2 degrades β-1,3-xylan into mainly β-1,3-XOs extracellularly (Fig. 2), which are subsequently transported into the cytoplasm for further catabolism. To date, no transporter for β-1,3-XOs has been identified. In strain EA2, *xul* encodes two classes of transporters, the MFS transporter XylT and the ABC transporter XylCDEF. XylT displays the highest amino acid similarity (31.5%) to XylP, the isoprimeverose transporter from *Lactobacillus pentosus* [43], and XylC (the substrate binding protein [SBP] of XylCDEF) (30.0%) to *Bl*AXBP, the β-1,4-XO-binding protein from *Bifidobacterium animalis* [44]. To determine the roles of XylT and XylCDEF in β-1,3-xylan catabolism, single and double gene deletion mutants (Δ*xylT*, Δ*xylC*, and Δ*xylT*Δ*xylC*) were constructed. A single gene deletion had some noticeable impact on the growth of strain EA2 on β-1,3-xylan, xylose, β-1,3X2, or β-1,3X3, and the double mutant Δ*xylT*Δ*xylC* almost completely lost its ability to grow on these substrates (Fig. 3). A complementation of both *xylT* and *xylC* in Δ*xylT*Δ*xylC* restored its growth to the WT level. These results suggest that both XylT and XylCDEF are capable of importing β-1,3-XOs. Comparatively, *xylC* deletion had a greater effect especially on the growth of strain EA2 on 2 mM β-1,3X2 and graded concentrations (2 mM, 50 μM, and 5 μM) of β-1,3X3 (Fig. 3C-F). Moreover, the transcriptional level of *xylC* was the most upregulated of all *xul* genes by β-1,3-xylan (Fig. 1D). These data suggest that XylCDEF plays a major role in β-1,3-XOs uptake under this experimental condition. However, the *in vivo* function of XylT and XylCDEF *in situ* needs further study.

**Figure 4:**
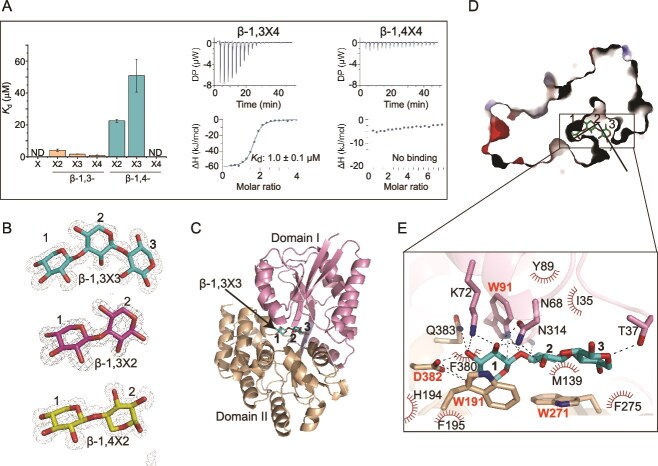
Biochemical and structural analyses of XylC, the substrate-binding protein of the ABC transporter XylCDEF. (A) Binding capacities of XylC to xylose (X) as well as β-1,3- and β-1,4-linked X2, X3, and X4. Representative ITC data for titrations of β-1,3X4 and β-1,4X4 into XylC were shown. The other ITC data are provided in Fig. S2. (B) *mFo-DFc* omit maps of β-1,3X3, β-1,3X2, and β-1,4X2. The simulated annealing *mFo-DFc* omit map was generated using the Phenix program. The resulting electron density map was contoured at 3σ in gray. (C) Overall structure of the XylC/β-1,3X3 complex. Domain I is colored in pink, and domain II in wheat. The β-1,3X3 molecule is shown as cyan sticks. (D) L-shaped substrate-binding pocket of XylC. The β-1,3X3 molecule is shown as cyan sticks. (E) Key residues of XylC interacting with β-1,3X3. Side chains of residues involved in hydrogen bonding and direct hydrophobic stacking interactions are shown as sticks, among which those from domain I are in pink and those from domain II are in wheat. Other hydrophobic residues are shown as red spoked arcs.

To further ascertain the function of XylT and XylCDEF, we attempted to biochemically characterize them *in vitro*. However, only XylC was successfully produced in *E. coli* (Fig. S1a) and unfortunately, the yield of the membrane protein XylT produced in *E. coli* was too low to be used for functional characterization. The substrate specificity of XylC was analyzed by testing its binding affinities to xylose as well as β-1,3- and β-1,4-linked X2, X3, and X4 using ITC (Fig. 4A and Fig. S2). Among all the tested XOs, XylC displayed the highest affinity to β-1,3X4 with a *K*_d_ (dissociation constant) value of 1.0 μM, but no binding to β-1,4X4. Its binding affinities to β-1,3X2 and β-1,3X3 were 5.5 times and 29.9 times that of β-1,4X2 and β-1,4X3, respectively, and no binding was detected to xylose. These results indicate that XylC prefers to bind β-1,3-XOs. We further compared the substrate specificity of XylC with that of the reported β-1,4-XO-binding proteins (similarity to XylC: ~30.0%), including *Bl*AXBP from *B. animalis* [44], XBP1 from *Caldanaerobius polysaccharolyticus* [45], and BxlE from *Streptomyces thermoviolaceus* [46]. The results showed that the β-1,4-XO-binding proteins showed no or negligible affinity to β-1,3-XOs (Table S4), significantly different from the binding preference of XylC to β-1,3-XOs.

### Structural basis for the binding preference of XylC to β-1,3-XOs

To reveal the mechanism by which XylC preferentially binds β-1,3-XOs, the crystal structures of XylC in complex with β-1,3X2, β-1,3X3, and β-1,4X2, separately, were solved (Table S5 and Fig. 4B). Xylose units were numbered according to the nomenclature of Ejby et al. [44]. The three structures are highly similar with root mean square deviations (RMSDs) of 0.18 ~ 0.23 Å. XylC comprises two domains (domains I and II) with the substrate bound between the two domains (Fig. 4C), similar to other SBPs of ABC transporters. A comparison using the Dali server [47] showed that the closely related structural homologs of XylC are β-1,4-XO-binding proteins, including *Bl*AXBP (PDB: 3ZKL; RMSD: 1.8 Å; Z-score: 51.5) [44], XBP1 (PDB: 4G68; RMSD: 2.0 Å; Z-score: 47.8) [45], and BxlE (PDB: 3VXC; RMSD: 2.1 Å; Z-score: 46.5) [46]. The binding pocket of XylC is L-shaped (Fig. 4D). Correspondingly, a kink of β-1,3X3 at site 2 was observed, which is exactly located at the “L” bend. In β-1,4-XO-binding proteins, however, the pockets are arc-shaped to accommodate the nearly linear β-1,4-XOs (Fig. S3a) [44–46]. In the structure of XylC-β-1,4X2, a ~ 45° deviation of xylose 2 was indeed observed compared to that in XylC-β-1,3X3 and XylC-β-1,3X2. The deviation results in the absence or increased distances of hydrogen bonds to Asn68, Trp91, and Asn314 (Fig. S3b), which explains the poor binding affinities of XylC to β-1,4X2.

Molecular details of substrate binding by XylC were further analyzed with the structure of XylC-β-1,3X3. By Ligplus [48] analysis, 16 residues were predicted to interact with β-1,3X3 by hydrogen bonding (Thr37, Asn68, Lys72, Trp91, Asn314, Asp382, and Gln383) or hydrophobic interactions (Ile35, Tyr89, Met139, Trp191, His194, Phe195, Trp271, Phe275, and Phe380) (Fig. 4E). Among the interactions, xylose 1 forms hydrogen bonds with Asn68, Lys72, Trp91, Asp382, and Gln383 and stacks directly onto Trp191. Xylose 2 forms hydrogen bonds with Asn68, Trp91, and Asn314 and stacks directly onto Trp271. Xylose 3 forms a hydrogen bond with Thr37. This binding mode shares little similarities with those reported in β-1,4-XO-binding proteins, except for interactions between the ligand and residues Asn68, Trp91, Trp191, Trp271, and Asp382 (Fig. S4). To further confirm the role of the 16 residues in substrate binding, alanine mutants of each residue were generated. ITC analysis showed that the binding affinities of W91A, W191A, W271A, and D382A to β-1,3X3 were completely abolished and those of the other mutants were decreased significantly (Fig. S5), indicating that among the 16 residues, Trp91, Trp191, Trp271, and Asp382 are essential whilst the others are also important for binding β-1,3X3. Circular dichroism (CD) spectroscopy analysis showed that the secondary structures of the mutants exhibit little deviation from that of WT XylC (Fig. S6), indicating that the binding affinities reduction was caused by amino acid replacement rather than by structural change.

Together, despite an overall structural similarity to β-1,4-XO-binding proteins [44–46], XylC has an L-shaped binding pocket, which is compatible with the conformation of β-1,3-XOs, but not with that of β-1,4-XOs. The substrate binding mode of XylC differs significantly from that of β-1,4-XO-binding proteins. Nevertheless, residues Trp91, Trp191, Trp271, and Asp382, whose site-directed mutagenesis incapacitates XylC, are relatively conserved in β-1,4-XO-binding proteins (Fig. S4), suggesting that the most crucial amino acid residues are preserved during protein evolution.

### XylRA positively regulates the transcription of *xul* with xylose being the effector

**Figure 5:**
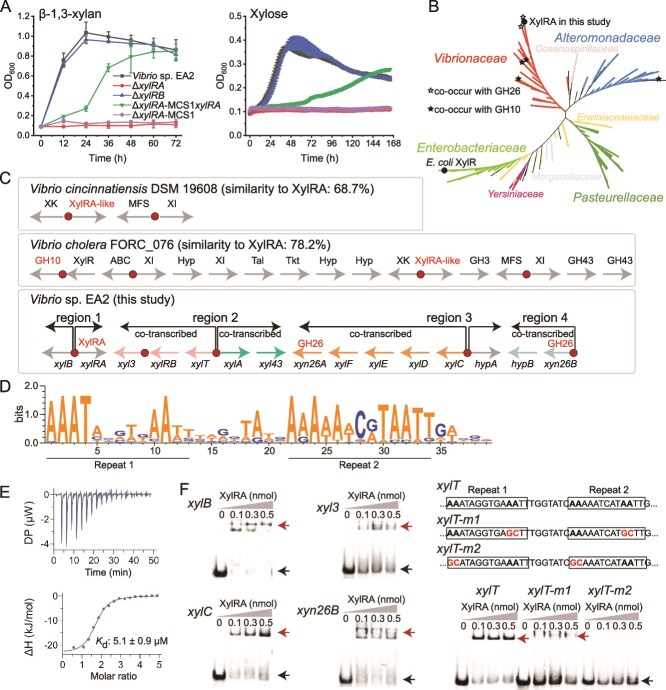
Analysis of the regulatory mechanism of XylRA on *xul*. (A) Growth curves of the wild-type strain EA2, the gene deletion mutants (Δ*xylRA* and Δ*xylRB*), the complemented mutant (Δ*xylRA*-MCS1*xylRA*), and the Δ*xylRA* mutant complemented with an empty plasmid (Δ*xylRA*-MCS1) on 0.2% (w/v) β-1,3-xylan and 5 mM xylose. (B) Distribution of XylRA homologs in bacteria. Branches are colored according to their taxonomic affiliations at the family level. XylRA proteins that co-occur with GH26 β-1,3-xylanases (in strain EA2 and an uncultured *Vibrio* strain) or GH10 β-1,4-xylanases (in *Vibrio* sp. Y2–5, *Vibrio hippocampi*, *Psychromonas marina*, and *Bowmanella yangjiangensis*) in an operon are marked with black stars. (C) XylRA-containing operons in three *Vibrio* genomes. The predicted XylRA binding sites are shown by red circles. DNA sequences of candidate XylRA binding sites in these and other four *Vibrio* genomes are listed in Table S6. RT-PCR results of co-transcription of *xul* genes in strain EA2 are provided in Fig. S8. (D) Sequence logo for the predicted XylRA binding sites in *Vibrio* strains. The logo was constructed using the predicted XylRA binding sites from seven *Vibrio* strains (Table S6). (E) Representative ITC data for titrations of xylose into recombinant XylRA. (F) EMSAs with recombinant XylRA and its target DNA binding sites. The DNA sequence of P*_xylT_* and its mutant sequences (P*_xylT-m1_* and P*_xylT-m2_*) are shown above their EMSAs. Black arrows indicate the free DNA and red arrows indicate the XylRA-DNA complexes. The data shown in the graphs are from triplicate experiments (mean ± SD or representative data).

In strain EA2, *xylRA* and *xylRB* were predicted to be transcriptional regulators in *xul* (Table S3), which display 61.7% and 26.0% similarities, respectively, to the activator XylR of xylose catabolism in *E. coli* [49]. Genetic analyses showed that the *xylRA*-deletion mutant strain (Δ*xylRA*) exhibited growth on neither β-1,3-xylan nor xylose, the *xylRB*-deletion mutant strain (Δ*xylRB*) displayed a similar growth to the WT strain EA2, and the growth of Δ*xylRA* was partially restored by *xylRA* complementation (Fig. 5A). These results suggest that it is likely that *xylRA*, rather than *xylRB*, positively regulates *xul*.

XylRA has a similar domain architecture to *E. coli* XylR [49], composed of an N-terminal ligand-binding domain of the LacI family and a C-terminal DNA-binding domain of the AraC family. A total of 258 homologs of XylRA were retrieved from NCBI clustered nr database (*E* value <1e^−50^, identity >50%). All these homologs are composed of LacI ligand-binding domain and AraC DNA-binding domain and largely retain the conserved sites necessary for binding xylose (Fig. S7a). The retrieved sequences are widely distributed in the class *Gammaproteobacteria*, mainly in families *Vibrionaceae*, *Alteromonadaceae*, *Enterobacteriaceae*, and *Pasteurellaceae* (Fig. 5B). In most cases (e.g. in *Vibrio cincinnatiensis* DSM 19608), *xylRA* co-occurs with genes involved in xylose metabolism, such as xylose isomerase and xylulokinase, constituting xylose utilization operons (Fig. 5B and 5C). However, it was also observed that *xylRA* co-occurs with genes encoding GH26 β-1,3-xylanases or GH10 β-1,4-xylanases in six bacterial strains, suggesting that in addition to regulating xylose metabolism, *xylRA* may also regulate the catabolism of β-1,3-xylan or β-1,4-xylan.

To investigate the mechanism by which XylRA regulates *xul*, we performed a cross-genomic comparison [50] and uncovered a conserved motif from XylRA-containing operons of strain EA2 and six additional *Vibrio* strains (Fig. 5D and Table S6). This motif contains two direct repeats rich in AT, resembling the DNA binding sites of XylR [49]. In *xul*, this putative XylRA binding motif was detected upstream *xylB* (P*_xylB_*), *xyl3* (P*_xyl3_*), *xylT* (P*_xylT_*), *xylC* (P*_xylC_*), and *xyn26B* (P*_xyn26B_*) (Fig. 5C and Table S6). We then tested whether XylRA interacts with these predicted DNA binding sites. XylRA was overproduced in *E. coli* and purified (Fig. S1a). ITC analysis showed that XylRA had a high binding affinity to xylose with a *K*_d_ value of 5.1 μM (Fig. 5E), but no affinity to β-1,3X2 or β-1,3X3 (data not shown), indicating that xylose is the effector for XylRA to activate *xul*. In the presence of xylose, binding of XylRA to biotin-labeled DNA probes (50 bp) containing P*_xylB_*, P*_xyl3_*, P*_xylT_*, P*_xylC_*, and P*_xyn26B_* were assessed by EMSA. The result showed that XylRA bound strongly with P*_xylB_*, P*_xylT_*, P*_xylC_*, and P*_xyn26B_*, but weakly with P*_xyl3_* (Fig. 5F). Based on gene orientations and co-transcriptional analyses, *xul* was divided into four regions (regions 1–4) (Figs. 5C and S8). The EMSA result suggests that XylRA regulates *xul* by mainly controlling the promoters of *xylB*, *xylT*, *xylC*, and *xyn26B*. When the conserved adenines (A) of P*_xylT_* were mutated into cytosines (C) or guanines (G), the binding was impaired (with the mutant P*_xylT-m1_*) or abolished (with the mutant P*_xylT-m2_*) (Fig. 5F), confirming that XylRA recognizes specific DNA sequences.

XylR is a regulator that activates xylose metabolism in *E. coli* [49]. Here, our results demonstrate that XylRA, a homolog of XylR, is a xylose-responsive activator regulating β-1,3-xylan catabolism in the *Vibrio* strain EA2. Moreover, XylRA homologs are also present in β-1,3-xylan or β-1,4-xylan utilization operons in other bacteria (Fig. 5B). This may reflect the rapid adaptive evolution of bacteria from xylose utilization to xylan utilization by HGTs. Many studies have shown that bacteria frequently adopt regulators that sense oligomers of two to eight monosaccharides in length [51–53]. In contrast, the regulatory strategy of polysaccharide catabolism by sensing monosaccharide is uncommon in bacteria and has only been reported in fructan catabolism by *Bacteroides thetaiotaomicron* [54], to our knowledge.

### Comparison of the β-1,3-xylan catabolism pathway in strain EA2 with those of β-1,4-xylan and other polysaccharides in other bacteria

**Figure 6:**
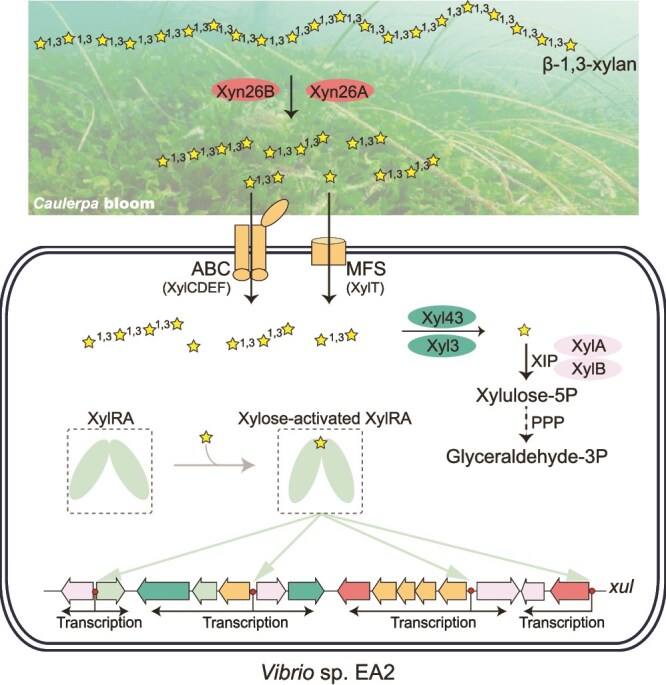
A schematic for the β-1,3-xylan catabolism pathway in strain EA2. Extracellularly, β-1,3-xylan is degraded by β-1,3-xylanases Xyn26A and Xyn26B into β-1,3-xylooligosaccharides, which are subsequently imported via the ABC transporter XylCDEF or the MFS transporter XylT. The β-1,3-xylooligosaccharides are completely saccharified by β-1,3-xylosidases Xyl43 and Xyl3. The produced xylose is metabolized through the xylose isomerase pathway (XIP) and the subsequent pentose phosphate pathway (PPP). XylRA is xylose-responsive to activate the transcription of *xul*. The binding sites of XylRA in *xul* are indicated with red solid circles. Black arrows represent the transcriptional directions of *xul* genes. Xylose molecules are indicated with yellow pentangles.

Based on the above results, we propose the β-1,3-xylan catabolism pathway adopted by strain EA2 (Fig. 6). This pathway includes: 1) extracellular decomposition of β-1,3-xylan into β-1,3-XOs by β-1,3-xylanases Xyn26A and Xyn26B; 2) import of the generated β-1,3-XOs by the master transporter XylCDEF and the candidate XylT; 3) intracellular decomposition of β-1,3-XOs into xylose by β-1,3-xylosidases Xyl3 and Xyl43, and xylose assimilation through the xylose isomerase pathway by the xylose isomerase XylA and the xylulokinase XylB. The pathway relies on the β-1,3-xylan utilization locus *xul*. XylRA activates the transcription of *xul* genes with xylose being the effector.

β-1,4-xylan, whose backbone is composed of β-1,4-linked xylose, is a major polysaccharide in the cell walls of terrestrial plants [38]. β-1,4-xylanolytic systems have been reported in two gut symbionts *Bacteroides ovatus* and *Prevotella bryantii* and the plant pathogen *Xanthomonas campestris* pv *campestris* [55–57]. In these bacteria, β-1,4-xylanases mediate the initial decomposition of β-1,4-xylan and the TonB-dependent transporters (TBDTs) are essential for β-1,4-XOs uptake. These systems are regulated by regulators of LacI family or hybrid two-component systems (HTCS). Therefore, the β-1,3-xylanolytic system in strain EA2 is different from the previously reported β-1,4-xylanolytic systems in transporters, regulators, and especially in enzyme systems. These analyses also suggest that bacteria catabolize a certain polysaccharide through a specialized system, even when coped with structurally similar polysaccharides.

In *Vibrio*, catabolism of alginate and chitin has been extensively investigated [51, 58–60]. A comparison with the β-1,3-xylanolytic system in strain EA2 revealed that, in addition to the enzymatic system, there are significant differences in both the transport system and the regulatory mechanism. Specifically, in the chitinolytic system, oligosaccharides are imported across the outer membrane via a specific porin (chitoporin), and across the inner membrane by an ABC transporter and a phosphotransferase system (PTS) transporter [58]. The chitinolytic system is regulated by a two-component system [51]. In the alginate utilization system, *Vibrio* employs an outer membrane porin (KdgMN) and an inner membrane symporter (ToaABC) for substrate transport [59]. Based on genomic analysis, the alginate pathway is regulated by LysR, GntR, IclR, or KdgR regulators [60]. Therefore, *Vibrio* seems to adopt different strategies for polysaccharide utilization depending on the polysaccharide type.

### Distribution of β-1,3-xylanases and β-1,3-xylan utilization loci in marine bacteria

**Figure 7:**
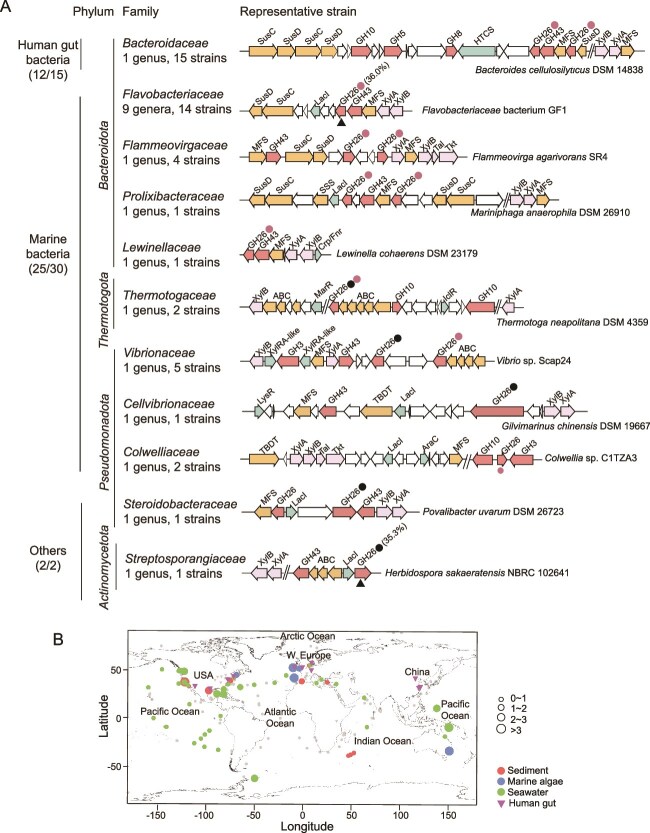
Distribution of β-1,3-xylanases and β-1,3-xylan utilization loci in bacteria. (A) Distribution of β-1,3-xylan utilization loci in bacterial genomes. With Xyn26A and Xyn26B, separately, as query sequences, a total of 47 bacterial isolates from 11 families that contain β-1,3-xylan utilization loci were identified. Red and black circles represent Xyn26A and Xyn26B homologs, respectively. Black triangles represent two randomly selected sequences for functional validation, which have ~35% identity (the lowest threshold) to the query sequence (similarities are shown in parentheses). (B) Distribution of Xyn26A and Xyn26B homologs in marine (sediments, algae, and seawater) and human gut metagenomes. Grey marks represent the sampling sites of all metagenomic datasets and colored ones represent those containing Xyn26A or Xyn26B homologs. The gene abundance at each marine sampling site was normalized and relative abundances were represented by circles with different sizes. HTCS, hybrid two-component system; MFS, major facilitator superfamily transporter; XylA, xylose isomerase; XylB, xylulokinase; LacI, LacI family regulator; Tal, transaldolase; Tkt, transketolase; Crp/Fnr, Crp/Fnr family regulator; MarR, MarR family regulator; IclR, IclR family regulator; XylRA-like, XylRA-like regulator; lysR, lysR family regulator; TBDT, TonB-dependent transporter; AraC, AraC family regulator.

To better understand the ecological significance of β-1,3-xylan catabolism in bacteria, the distribution of β-1,3-xylanase genes in bacterial genomes was investigated with Xyn26A and Xyn26B, separately, as query sequences in IMG database. A total of 126 homologs (*E* value <1e^−50^, identity >35%) were obtained. These homologs largely retain the conserved sites necessary for β-1,3-xylan binding and catalysis (Fig. S7b), suggesting that they are likely functional β-1,3-xylanases. To support this, we expressed two randomly selected sequences near the lowest threshold (similarities to the query sequence: 35.3% and 36.0%) and found that their recombinant products showed 168.5 ± 14.1 U/μmol and 30.4 ± 2.3 U/μmol activity on β-1,3-xylan (Fig. 7A). These analyses indicate that the cut-off value of identity >35% for screening is reasonable. The retrieved sequences are predominantly distributed in families *Flavobacteriaceae* (33.3%), *Bacteroidaceae* (27.0%), *Vibrionaceae* (10.3%), and *Flammeovirgaceae* (7.9%) (Fig. S9a and Table S7).

After manual curation of each genome, we identified 47 strains containing β-1,3-xylan utilization loci (Table S8). These strains belong to 11 families of phyla *Bacteroidota* (38 strains), *Pseudomonadota* (9 strains), *Actinomycetota* (1 strain), and *Thermotogota* (2 strains), most of which are either marine bacteria (25/47) or human gut symbionts (12/47) (Fig. 7A). Almost all the loci (except two lacking β-1,3-xylosidase genes) contain all genes encoding key enzymes for β-1,3-xylan utilization, including GH26 β-1,3-xylanases, GH3 or GH43 β-1,3-xylosidases, xylose isomerase XylA, and xylulokinase XylB (Fig. S9b), suggesting that there is a complete enzymatic system to utilize β-1,3-xylan in these strains, as that in strain EA2. A further comparison revealed that the gene compositions of the loci in strains from the same family are relatively conserved. For example, β-1,3-xylan utilization loci found in five *Vibrio* strains are analogous to *xul* despite some evolutionary events (e.g. the duplication of ABC transporter genes) (Table S8). However, the compositions are variable between different phyla or between different families of the same phyla, notably in transporters and regulators (Fig. 7A). An exception is that in *Bacteroidota*, the *susC/D* pairs, hallmarks of the *Bacteroidota* polysaccharide utilization loci [39], are well conserved.

We probed metagenomes of marine ecosystems, and normalized gene abundances were used to determine the prevalence of β-1,3-xylanase genes. These metagenomes include datasets of seawater, sediments, and algae, obtained from global oceans (Fig. 7B). Many datasets from the Pacific Ocean and the North Atlantic Ocean contain β-1,3-xylanase genes. Significantly higher gene abundances were found in datasets from coastal sites, reflecting the distribution of β-1,3-xylan-containing algae in coastal marine environments.

Together, β-1,3-xylanases and β-1,3-xylan utilization loci are widely distributed in diverse marine bacteria, predominantly in *Bacteroidota* and *Pseudomonadota*. β-1,3-xylan utilization loci in *Bacteroidota* are conserved but variable in other phyla, which may be the result of multiple evolutionary events including gene duplications and HGTs. Previous studies have demonstrated utilization pathways for a variety of algal polysaccharides in marine bacteria [4, 40, 61, 62], highlighting the important role of bacterial catabolism of algal polysaccharides in marine carbon cycle. Our revelation of the complete pathway of β-1,3-xylan catabolism by marine bacteria with strain EA2 as a model bacterium offers a mechanistic insight into the turnover of β-1,3-xylan, an important part of the marine carbon cycle.

**Figure 8:**
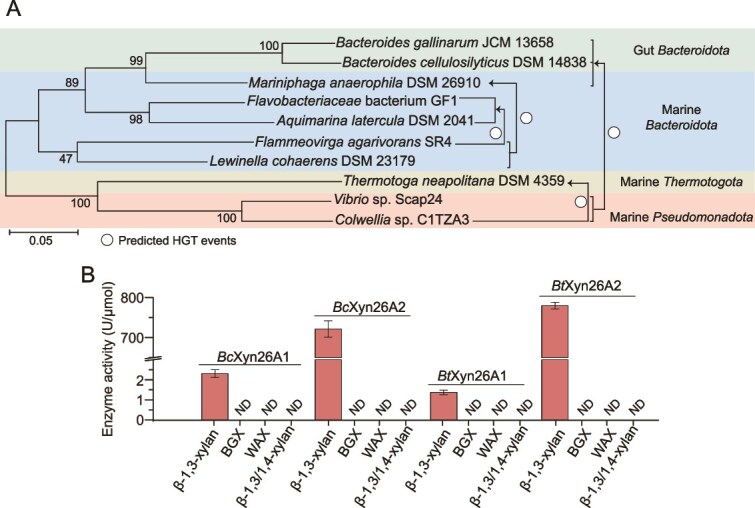
Transfer of β-1,3-xylanases from marine bacteria to human gut microbiota. (A) Horizontal gene transfer events of β-1,3-xylanases predicted by RANGER-DTL 2.0. The neighbor-joining tree was built based on the 16S rRNA gene sequences of selected marine and human gut bacteria containing Xyn26A homologs. (B) Functional validation of Xyn26A homologs in human gut microbiota. Four Xyn26A homologs from human gut bacteria *Bacteroides cellulosilyticus* DSM 14838 and *Bacteroides timonensis* AP1 were selected for substrate specificity analysis at 40°C in PBS (pH 7.0). BGX, beechwood glucuronoxylan; WAX, wheat arabinoxylan. Data shown in the graph are from triplicate experiments (mean ± S.D.).

### Transfer of β-1,3-xylanases from marine bacteria to human gut microbiota

The presence of GH26 genes in human gut *Bacteroidota* (Fig. 7A) called our attention for potential HGTs. Using RANGER-DTL [36], HGTs were inferred by reconciling a GH26 gene tree and a corresponding species tree (Fig. 8A). The result showed that GH26 genes were transferred from marine *Pseudomonadota* to other phyla, as well as between marine *Bacteroidota* (Fig. 8A). Thus, the GH26 genes in human gut *Bacteroidota* likely have a marine origin. To determine whether these gene products from human gut microbiota can function as β-1,3-xylanases, we selected four GH26 genes from two human gut bacteria, *Bacteroides cellulosilyticus* DSM 14838 (*bcXyn26A1* and *bcXyn26A2*) and *Bacteroides timonensis* AP1 (*btXyn26A1* and *btXyn26A2*), and overexpressed them in *E. coli*. The four recombinant enzymes were all robust β-1,3-xylanases, degrading only β-1,3-xylan among the tested xylans (Fig. 8B), which suggested that some human gut bacteria might have gained the ability to degrade β-1,3-xylan by recruiting β-1,3-xylanases from marine bacteria. In addition, β-1,3-xylanases were detected in the metagenomes of human gut near the coasts of the United States, China, and Western Europe (Fig. 7B).

Many gut bacteria are equipped with the ability to digest dietary polysaccharides. β-1,3-xylan is a dietary polysaccharide found in many edible algae, such as *Caulerpa* and *Porphyra* species. The β-1,3-xylan utilization loci in human gut *Bacteroidota* may be acquired by contact with edible algae and associated marine bacteria. Similar evolutionary events have been previously demonstrated in examples in which human gut bacteria acquire genes from marine bacteria for degrading algal porphyrin [63], agarose [64], and alginate [65]. Our results provide another example that genes degrading algal polysaccharides can be transferred from marine bacteria to human gut microbiota and support the notion that such transfers are extensive, as raised by Pudlo et al. [66]. This knowledge will help us understand the adaptations of the gut microbiome to human diet. However, how β-1,3-xylanases transfer from marine bacteria to the human gut microbiota still needs further investigation.

## Conclusion

Here, we show the complete pathway of β-1,3-xylan utilization adopted by the marine bacterium *Vibrio* sp. strain EA2, including the enzymatic and transport systems, and its regulation (Fig. 6). This pathway is carried out by a complex β-1,3-xylan utilization locus, *xul*. Such β-1,3-xylan utilization loci are widely distributed in marine bacteria, predominantly in *Bacteroidota* and *Pseudomonadota*, indicating the universality of β-1,3-xylan to be catabolized by marine bacteria as a carbon source, which may play a yet unappreciated, but important, role in the marine carbon cycle. Moreover, β-1,3-xylanases have transferred into human gut microbiota. The results shed light on how microbes utilize β-1,3-xylan and set a foundation for the metabolic engineering of marine *Vibrio* into platform organisms for β-1,3-xylan degradation.

## Supplementary Material

Supplementary_Materials_wraf085

Table_S1_wraf085

Table_S6_wraf085

Table_S7_wraf085

Table_S8_wraf085

## Data Availability

The genomic data of strain EA2 are available in NCBI under the accession number ASM3304249v1. The structures of XylC/β-1,3X2, XylC/β-1,3X3, and XylC/β-1,4X2 have been deposited in the PDB under the accession codes 8XBB, 8XBC, and 8XBA, respectively.
