# Supplementary material for: Complete xylan utilization pathway and regulation mechanisms involved in marine algae degradation by cosmopolitan marine and human gut microbiota
Source: ISME J. 2025 May 22;19(1):wraf085. doi: 10.1093/ismejo/wraf085 (PMC12125979; doi:10.1093/ismejo/wraf085)
Supplement: Supplementary_Materials_wraf085 [file supplementary_materials_wraf085.pdf]

Supplementary Materials for

Complete xylan utilization pathway and regulation mechanisms involved in marine algae degradation by cosmopolitan marine and human gut microbiota

Hai-Ning Sun[1#], Xiu-Lan Chen[1#]*, Yan Wang[1], Yan-Ping Zhu[1], Zhao-Jie Teng[1], Hai-Yan Cao[1,2], Ting-Ting Xu[1], Yin Chen[2,3], Yu-Zhong Zhang[2,4,5]*, Fang Zhao[1]*

[1]State Key Laboratory of Microbial Technology, Shandong University, Qingdao, China

[2]MOE Key Laboratory of Evolution and Marine Biodiversity, Frontiers Science Center for Deep Ocean Multispheres and Earth System & College of Marine Life Sciences, Ocean University of China, Qingdao, China

[3]School of Life Sciences, University of Warwick, Coventry, United Kingdom

[4]Laboratory for Marine Biology and Biotechnology, Qingdao Marine Science and Technology Center & Laoshan Laboratory, Qingdao, China

[5]Marine Biotechnology Research Center, State Key Laboratory of Microbial Technology, Shandong University, Qingdao, China

[#]Xiu-Lan Chen and Hai-Ning Sun contributed equally to this work.

[*]**Address correspondence to**: Yu-Zhong Zhang, zhangyz@sdu.edu.cn; Xiu-Lan Chen, cxl0423@sdu.edu.cn; Fang Zhao, zfang992@163.com

The authors declare no competing interests.

This PDF includes supplementary materials and methods, Tables S2-S5, Figures S1-S9. Tables S1 and S6-S8 are provided as separate .xlsx files.

Supplementary Materials and Methods

Growth experiments. When cells were cultivated on carbon sources at 0.2% (w/v), 5 mM and 2 mM concentrations (as indicated in the corresponding figure legends), the washed cells were diluted to $OD_{600} \approx 1.0$ followed by inoculation with 1% (v/v) of this cell suspension. Cultivation was conducted at 30°C and cell densities were monitored by OD_{600} using a spectrophotometer V-550 (Jasco Corporation, Japan). When cells were cultivated on 50 µM and 5 µM β-1,3-xylotriose, the washed cells were diluted to cell density $\approx 5 \times 10^4$ cells/mL followed by inoculation with 1% (v/v) of this cell suspension. Cultivation was conducted at 30°C and cell densities were monitored by flow cytometry using a Guava EasyCyte HT flow cytometer (Millipore, USA). The instrument was configured as follows: channel 01 for bright field imaging; channel 02 for fluorescence detection (Green-B fluorescence with an excitation wavelength of 488 nm); and the flow rate was set to low speed. Prior to flow cytometric analysis, cells were stained with SYBR Green I (Shanghai yuanye Bio-Technology, China) for 40 min at room temperature in the dark.

RT-qPCR. Total RNA was extracted using the RNeasy Mini Kit (Qiagen, Germany). Reverse transcription was performed by using the TransScript all-in-one First-Strand cDNA Synthesis SuperMix (TransGen, China). The RT-qPCR was performed on the LightCycler II 480 System (Roche, Switzerland) with SYBR Premix Ex Taq (TaKaRa, Japan). Data were analyzed by the $2^{-\Delta\Delta Ct}$ method [1] with the LightCycler 480 software. The *recA* gene was used as an internal reference.

Genetic manipulation. The upstream and downstream (~0.5 kb) of genes were

amplified from the genomic DNA of strain EA2. The two DNA fragments were joined by overlapping PCR and ligated into the *SalI* and *SacI* sites of the suicide plasmid pDM4 [2]. The constructed vector was then conjugated into strain EA2 to generate mutant strains. Single crossover strains were screened on MB plates containing both gentamycin (7.5 μg/mL) and chloramphenicol (25 μg/mL). Double crossover strains were screened on MB plates containing 12% (w/v) L-sucrose. For the construction of the complemented strains, the target genes and the corresponding promoter regions (0.2-1.0 kb) were cloned into plasmid pBBR1-MCS1 (Miaoling biology, China). The resulting plasmids were transformed into *E. coli* SM10 λpir and then conjugated into the gene knockout mutants [2].

Gene cloning, site-directed mutagenesis, protein production and purification. Genes encoding Xyn26A, Xyn26B, Xyl3, Xyl43, XylA, XylB, XylC, and XylRA were amplified from the genomic DNA of strain EA2 using PCR. The primers used are listed in Table S1. Genes encoding Xyn26A homologs, including *Bc*Xyn26A1 (IMG: 644168260) and *Bc*Xyn26A2 (IMG: 644168263) from *Bacteroides cellulosilyticus* DSM 14838, as well as *Bt*Xyn26A1 (IMG: 2600893258) and *Bt*Xyn26A2 (IMG: 2600893261) from *Bacteroides timonensis* AP1, were synthesized by the Beijing Genomics Institute (China). Genes were inserted into the *NdeI*/*XhoI* sites of pET-22b (Novagen, Germany) with a C-terminal His tag using an In-Fusion HD cloning kit (TaKaRa, Japan). The constructed expression plasmids were transformed into *E. coli* BL21 (DE3). The cells were cultivated at 37°C in the lysogeny broth (LB) medium containing 0.1 mg/mL ampicillin to OD_{600}≈0.8 and then induced with 0.5 mM

isopropyl-β-D-thiogalactopyranoside (IPTG) at 18°C for 18 h. The cells were harvested by centrifugation (9,000 *g* at 4°C for 5 min), resuspended in the lysis buffer (50 mM Tris-HCl, 100 mM NaCl, 0.5% glycerol, pH 8.0), and lysed by a pressure crusher. The lysate was centrifuged (13,000 *g* at 4°C for 60 min). The target protein in the supernatant was purified by nickel affinity chromatography on a Ni^{2+}-NTA column (GE Healthcare, USA), followed by desalination on PD-10 desalting columns (GE Healthcare, USA). Purified proteins were stored in 10 mM Tris-HCl buffer (pH 8.0) containing 100 mM NaCl at -80°C for further use. Protein purities were determined with SDS-PAGE. Protein concentrations were determined by a bicinchoninic acid protein assay kit (Thermo, USA) with bovine serum albumin as the standard.

Enzyme assays and characterization. Unless otherwise noted, enzyme assays were performed in PBS (20 mM) under their respective optimum pHs and temperatures. For DNS assays [3], reaction mixtures contained 10 μL β-1,3-xylanase solution (2.6-153.7 μM) and 90 μL xylan substrate (10 mg/mL). After 10 min incubation, reactions were terminated by adding 200 μL DNS. Next, samples were boiled for 5 min and OD_{550} was measured. The standard curve was generated with xylose (0-5.0 mM). One unit of enzyme activity (U) was defined as the amount of enzyme required to release 1 μmol xylose per min. With pNPX as the substrate for β-1,3-xylosidases, the reaction mixture contained 10 μL β-1,3-xylosidase solution (0.3-8.3 μM) and 90 μL pNPX (1 mM). After 10 min incubation, 600 μL Na_2CO_3 (1 M) was added, and the p-nitrophenyl (pNP) absorbance at 405 nm was measured. The standard curve was generated with pNP (0-0.7 mM). One U was defined as the amount of enzyme required to release 1 μmol pNP

per min. With XOs as the substrates for β-1,3-xylosidases, enzyme activities were determined by monitoring the produced xylose with a D-xylose assay kit (Megazyme, Ireland) [4]. The reaction mixture contained 10 μL β-1,3-xylosidase solution (0.004-83.2 μM) and a 90 μL mixture of NAD^+, ATP, hexokinase, xylose dehydrogenase, xylose mutarotase, and XOs substrates (2 mM) [4]. After 10 min incubation, reactions were terminated by 150 μL Na_2CO_3 (1 M) and the NADH absorbance at 340 nm of the mixture was measured. The standard curve was generated with xylose (0-0.8 mM). One U was defined as the amount of enzyme required to release 1 μmol xylose per min. To assay the activity of xylose isomerase [5], reaction mixtures contained 10 μL xylose isomerase (~0.55 μM) and a 90 μL mixture of $MgSO_4$ (5 mM) and xylose (5 mM). After 10 min incubation, 20 μL cysteine hydrochloride (1.5% [w/v]), 600 μL sulfuric acid (70% [v/v]), and 20 μL carbazole (0.12% [w/v]) were immediately added for color development, and OD_{540} was measured. The standard curve was generated with xylulose (0-0.25 mM). One U was defined as the amount of enzyme required to release 1 μmol xylulose per min. To assay xylulokinase activity, the 100 μL reaction mixture contained xylulokinase (~0.03 μM), $MgCl_2$ (2 mM), NADH (1 mM), xylulose (5 mM), phosphoenolpyruvate (0.2 mM), pyruvate kinase (1 U), lactate dehydrogenase (1 U), and ATP (2 mM) [6]. After 10 min incubation, reactions were terminated by 150 μL Na_2CO_3 (1 M) and the NADH absorbance at 340 nm was measured. The standard curve was generated with NADH (0-1.0 mM). One U was defined as the amount of enzyme that oxidizes 1 μmol NADH to NAD^+ per min.

Enzyme characterization was performed with β-1,3-xylan, pNPX, xylose and

xylulose as the substrate, respectively, for β-1,3-xylanases (Xyn26A and Xyn26B), β-1,3-xylosidases (Xyl3 and Xyl43), xylose isomerase XylA and xylulokinase XylB. Effect of temperature was determined in PBS from 10°C to 60°C at an interval of 10°C. Effect of pH was determined in Britton-Robinson buffer with pH values from pH 3.0 to 11.0. To analyze the products of β-1,3-xylanases, reaction mixtures containing 20 µL β-1,3-xylanase solution and 180 µL β-1,3-xylan (10 mg/mL) were incubated at 30°C. At different time points within 24 h, products were analyzed using size-exclusion chromatography on a Superdex 30 Increase 10/300 GL column (GE Healthcare, USA) [7]. To analyze the products of XylA and XylB catalyzing xylose, a 100 µL reaction mixture containing XylA (~5 µM), XylB (~5 µM), xylose (10 mM), $MgCl_2$ (2 mM), and ATP (2 mM) in the Tris-HCl buffer (pH 7.0; 5 mM) was incubated at 30°C for 24 h. After incubation, the reaction products were analyzed by Q-TOF-MS from *m/z* 50 to 1,500 in a negative ion mode.

Isothermal titration calorimetry (ITC). Concentrations of proteins XylC and XylRA were 40 µM and 80 µM, respectively. Ligands used were 20-100 times the concentration of corresponding proteins. Each ligand was injected into the protein cell 19 times with a stirring speed of 750 rpm.

Crystallization, data collection and structure determination. The purified XylC was concentrated to 10 mg/mL in 10 mM Tris- HCl (pH 8.0) containing 100 mM NaCl. Before crystallization, XylC was mixed with β-1,3X2, β-1,3X3, or β-1,4X2 (2 mM) on ice for 1 h. Initial crystallization trials were performed at 18°C using the sitting-drop vapor diffusion method and diffraction-quality crystals were obtained using the hanging-drop vapor-diffusion method. XylC/β-1,3X2 was obtained in hanging drops containing 1 M lithium chloride, 0.1 M citric acid (pH 4.0), and 20% (w/v) polyethylene glycol (PEG) 6000. XylC/β-1,3X3 was obtained in handing drops containing 2 M

ammonium sulfate dibasic, 0.1 M sodium acetate/acetic acid, and 2% (w/v) PEG 400. XylC/β-1,4X2 was obtained in hanging drops containing 0.2 M ammonium chloride and 20% (w/v) PEG 3350. X-ray diffraction data were collected at Shanghai Synchrotron Radiation Facility. The initial diffraction data sets were processed using the HKL3000 program [8]. Crystal structures were determined by molecular replacement using the CCP4 program Phaser [9]. The structure of XylC predicted by Alpha-Fold2 [10] was used as the search model. Structure refinement was performed using Coot [11] and Phenix [12]. All the structure figures were processed using the program PyMOL.

Circular dichroism (CD) spectroscopy. CD spectra for XylC and its mutants were carried out on a JASCO J-1500 Spectrometer (Japan). All proteins were adjusted to a final concentration of 0.3 mg/mL in 10 mM Tris-HCl (pH 8.0) containing 100 mM NaCl. Spectra were recorded from 250 to 200 nm at a scan speed of 200 nm/min.

Reverse transcription PCR (RT-PCR). RT-PCR was used to analyze co-transcriptional relationships of *xul* genes. The cells of strain EA2 cultivated with xylose as the sole carbon source were used for DNA extraction, RNA extraction, and cDNA synthesis. PCR was performed with cDNA, DNA (used as the positive control), and RNA (used as the negative control) as templates. The primers used are listed in Table S1. Each primer pair spanned the intergenic region between two ORFs.

Electrophoretic mobility shift assay (EMSA). DNA probes (50 bp) containing the predicted XylRA binding sites from the promoter regions of *xylB*, *xyl3*, *xylT*, *xylC*, and *xyn26B* were synthesized and labeled with 5'-Biotin by Tsingke (China). DNA probes (25 ng) were mixed with recombinant XylRA (0.1-0.5 nmol) in 20 μL EMSA/Gel-shift binding buffer (Beyotime, China). The mixture was incubated at room temperature for 20 min, followed by separation on a 10% precast-Glgel TBE PAGE (Sangon Biotech,

China) in an ice bath. Next, DNA probes and proteins were transferred from the gel to a positively charged nylon membrane, fixed, blocked, washed, and finally stained using solutions of an ECL Western blotting kit (Absin, China).

Bioinformatics. To explore the distribution of XylRA in bacteria, XylRA was used as the query sequence to extract homologs from NCBI clustered nr database with a cut-off value of E value < $1e^{-50}$, identity > 50% and coverage > 75%. The cut-off value was scrutinized by multiple sequence alignment of retrieved sequences using MEGA X [13]. Based on the alignment result, domain architectures of retrieved sequences and key residues involved in binding xylose were further analyzed according to the structure of XylR (PDB: 4FE7) [14]. Operons containing XylRA homologs were manually checked. Conserved DNA motifs were searched in all promoter regions of XylRA-containing operons from strain EA2 and six additional *Vibrio* strains (Table S6), using MEME (the Multiple EM for Motif Elicitation; http://memesuite.org/), with a motif site distribution of zero or one site per sequence and a motif width > 10 and < 50. Sequence logos were drawn with WebLogo 3 (http://weblogo.threeplusone.com).

To explore the distribution of β-1,3-xylanases in bacteria, catalytic domains of Xyn26A (M1-D350) and Xyn26B (M1-G350) were used as query sequences to retrieve homologs from IMG [7]. To retrieve homologs from bacterial isolates, all available bacterial genomes in IMG were probed, with a cut-off value of E value < $1e^{-50}$ and identity > 35%. The cut-off value was scrutinized by multiple sequence alignment of retrieved sequences using MEGA X [13] and by analyzing key residues involved in substrate-binding and catalysis according to the structure of Xyl4 (PDB: 3VPL) [15]. To further validate the function of retrieved sequences, one Xyn26A homolog from *Flavobacteriaceae* bacterium GF1 (IMG accession no. 2909472311) and one Xyn26B homolog from *Herbidospora sakaeratensis* NBRC 102641 (IMG accession no.

2725998680), which have ~35% similarity (the lowest threshold) to the query sequence, were overexpressed in *E. coli* BL21 (DE3) and purified, and their activities were determined with β-1,3-xylan as the substrate at 40°C. To retrieve homologs of Xyn26A and/or Xyn26B from metagenomes, a total of 3,521 metagenomes were chosen from IMG, including 1,372 from seawater, 369 from marine sediments, 50 from marine algae, and 1,730 from human guts. A BlastP analysis was then performed against these metagenomes. Gene reads were normalized by dividing the retrieved read by gene length (kb) and gene abundances were normalized by dividing the normalized gene reads by genome size (Mb).

References

1. Pfaffl MW. A new mathematical model for relative quantification in real-time RT-PCR. Nucleic Acids Res. 2001;29:e45
2. Zhang J, Liu B, Gu D, Hao Y, Chen M, Ma Y et al. Binding site profiles and N-terminal minor groove interactions of the master quorum-sensing regulator LuxR enable flexible control of gene activation and repression. Nucleic Acids Research. 2021;49:3274-93
3. Miller GL, Blum R, Glennon WE, Burton AL. Measurement of carboxymethylcellulase activity. Anal Biochem. 1960;1:127-32
4. Zhao F, Yu CM, Sun HN, Xu TT, Sun ZZ, Qin QL et al. The catabolic specialization of the marine bacterium *Polaribacter* sp. Q13 to red algal β1,3/1,4-mixed-linkage xylan. Appl Environ Microbiol. 2024;90:e0170423
5. Dische Z, Borenfreund E. A new spectrophotometric method for the detection and determination of keto sugars and trioses. J Biol Chem. 1951;192:583-87
6. Bu S, Tsang PWK, Fu RZ. GroEL-GroES solubilizes abundantly expressed xylulokinase in *Escherichia coli*. J Appl Microbiol. 2005;98:210-15
7. Sun HN, Yu CM, Fu HH, Wang P, Fang ZG, Zhang YZ et al. Diversity of marine 1,3-xylan-utilizing bacteria and characters of their extracellular 1,3-xylanases. Front Microbiol. 2021;12:721422
8. Minor W, Cymborowski M, Otwinowski Z, Chruszcz M. HKL-3000: The integration of data reduction and structure solution-from diffraction images to an initial model in minutes. Acta Crystallogr D Biol Crystallogr. 2006;62:859-66
9. Winn MD, Ballard CC, Cowtan KD, Dodson EJ, Emsley P, Evans PR et al. Overview of the CCP4 suite and current developments. Acta Crystallogr D Biol Crystallogr. 2011;67:235-42
10. Jumper J, Evans R, Pritzel A, Green T, Figurnov M, Ronneberger O et al. Highly accurate protein structure prediction with AlphaFold. Nature. 2021;596:583-89
11. Emsley P, Lohkamp B, Scott WG, Cowtan K. Features and development of Coot. Acta Crystallogr D Biol Crystallogr. 2010;66:486-501
12. Adams PD, Afonine PV, Bunkóczi G, Chen VB, Davis IW, Echols N et al. PHENIX: A

comprehensive Python-based system for macromolecular structure solution. Acta Crystallogr D Biol Crystallogr. 2010;66:213-21

13. Kumar S, Stecher G, Li M, Knyaz C, Tamura K. MEGA X: Molecular evolutionary genetics analysis across computing platforms. Mol Biol Evol. 2018;35:1547-49

14. Ni L, Tonthat NK, Chinnam N, Schumacher MA. Structures of the *Escherichia coli* transcription activator and regulator of diauxie, XylR: An AraC DNA-binding family member with a LacI/GalR ligand-binding domain. Nucleic Acids Res. 2013;41:1998-2008

15. Goddard-Borger ED, Sakaguchi K, Reitinger S, Watanabe N, Ito M, Withers SG. Mechanistic insights into the 1,3-xylanases: Useful enzymes for manipulation of algal biomass. J Am Chem Soc. 2012;134:3895-902

Table S1 (a separate .xlsx file) Primers used in this study.

Table S2 Strains and plasmids used in the genetic manipulations of strain EA2.

Strains or plasmids	Phenotype and genotype
Strains	
E. coli DH5α λpir [a]	Host for π-requiring plasmids
E. coli SM10 λpir [a]	Host for π-requiring plasmids, conjugal donor, Km^r
Δ*xylRA*	*xylRA* gene deletion mutant of strain EA2
Δ*xylRB*	*xylRB* gene deletion mutant of strain EA2
Δ*xylT*	*xylT* gene deletion mutant of strain EA2
Δ*xylC*	*xylC* gene deletion mutant of strain EA2
Δ*xylT*Δ*xylC*	Double gene deletion mutant of *xylT* and *xylC* of strain EA2
Δ*xylRA*-MCS1*xylRA*	The complementary strain of Δ*xylRA*
Δ*xylRA*-MCS1	Control strain of Δ*xylRA* carrying the empty plasmid pBBR1-MCS1
Δ*xylT*Δ*xylC*-MCS1*xylTxylC*	The complementary strain of Δ*xylT*Δ*xylC*
Δ*xylT*Δ*xylC*-MCS1	Control strain of Δ*xylT*Δ*xylC* carrying the empty plasmid pBBR1-MCS1
Δ*xyn26A*	*xyl26A* gene deletion mutant of strain EA2
Δ*xyn26B*	*xyl26B* gene deletion mutant of strain EA2
Δ*xyl3*	*xyl3* gene deletion mutant of strain EA2
Δ*xyl43*	*xyl43* gene deletion mutant of strain EA2
Δ*xylA*	*xylA* gene deletion mutant of strain EA2
Δ*xylB*	*xylB* gene deletion mutant of strain EA2
Δ*xyl3*-MCS1*xyl3*	The complementary strain of Δ*xyl3*
Δ*xyl43*-MCS1*xyl43*	The complementary strain of Δ*xyl43*
Δ*xylA*-MCS1*xylA*	The complementary strain of Δ*xylA*
Δ*xylB*-MCS1*xylB*	The complementary strain of Δ*xylB*
Δ*xyl3*-MCS1	Control strain of Δ*xyl3* carrying pBBR1-MCS1
Δ*xyl43*-MCS1	Control strain of Δ*xyl43* carrying pBBR1-MCS1
Δ*xylA*-MCS1	Control strain of Δ*xylA* carrying pBBR1-MCS1
Δ*xylB*-MCS1	Control strain of Δ*xylB* carrying pBBR1-MCS1
Plasmids	
pDM4 [a]	Suicide vector for gene knockout in strain EA2
pBBR1-MCS1	Plasmid for complementing knockout mutants in strain EA2

[a] A gift from Prof. Qi-Yao Wang (East China University of Technology).

Table S3 Functional annotations of *xul*-encoded proteins[a].

Abbreviation	Locus tag	Length (aa) / SP	Representative homolog (NCBI accession)	Identity (%) / Coverage (%)	Predicted annotation in strain EA2
XylB	RXX31_21090	483 / No	Xylulokinase (P09099.1)	64.9% / 100%	Xylulokinase
XylRA	RXX31_21095	430 / No	LacI/AraC regulator (P0ACI3.1)	61.7% / 91%	Regulatory protein
Xyl3	RXX31_21105	788 / No	β-xylosidase (T2KMH0.1)	35.3% / 84%	Xylosidase, GH3
XylRB	RXX31_21110	370 / No	LacI/AraC regulator (P0ACI3.1)	26.0 % / 97%	Regulatory protein
XylT	RXX31_21115	462 / No	MFS transporter (P75683.1)	31.5% / 98%	Xyloside transporter, MFS superfamily
XylA	RXX31_21120	439 / No	Xylose isomerase (BAI23199.1)	88.2% / 100%	Xylose isomerase
Xyl43	RXX31_21125	538 / No	β-1,3-xylosidase (A9ZND1.1)	80.8% / 99%	Xylosidase, GH43
Xyn26A	RXX31_21135	488 / Yes	β-1,3-xylanase (Q9LCB9.1)	30.6% / 68%	β-1,3-xylanase, GH26
XylF	RXX31_21140	365 / No	ATP-binding protein (Q9Z3R9.2)	52.6% / 98%	ABC transporter, ATP-binding protein
XylE	RXX31_21145	275 / No	Permease protein (O50501.1)	31.8% / 95%	ABC transporter, permease protein
XylD	RXX31_21150	316 / No	Permease protein (O50500.1)	29.4% / 86%	ABC transporter, permease protein
XylC	RXX31_21155	421 / Yes	Isoprimeverose transporter (O32156.1)	30.0% / 94%	ABC transporter, substrate binding protein
HypA	RXX31_21160	678 / No	--	--	Hypothetical protein
HypB	RXX31_21165	347 / No	--	--	Hypothetical protein
Xyn26B	RXX31_21170	582 / Yes	β-1,3-xylanase (D5MP61.1)	95.6% / 100%	β-1,3-xylanase, GH26

[a] Signal peptides (SP) were predicted by SignalP 5.0.

Table S4 Binding capacities of β-1,4XO-binding proteins to xylose as well as β-1,3- and β-1,4-linked X2, X3, and X4[a].

Protein	Substrate	K_d values
BxlE	Xylose	ND
	β-1,4X2	15.1 ± 4.04 nM
	β-1,4X3	6.28 ± 1.93 nM
	β-1,4X4	13.2 ± 1.93 nM
	β-1,3X2	ND
	β-1,3X3	ND
	β-1,3X4	ND
*Bl*AXBP	Xylose	ND
	β-1,4X2	4.92 ± 1.27 μM
	β-1,4X3	49.8 ± 9.7 nM
	β-1,4X4	30.5 ± 2.05 nM
	β-1,3X2	ND
	β-1,3X3	ND
	β-1,3X4	ND
XBP1	Xylose	ND
	β-1,4X2	47.3 ± 15.5 nM
	β-1,4X3	23.5 ± 7.0 nM
	β-1,4X4	82.8 ± 11.2 nM
	β-1,3X2	23.0 ± 14.4 μM
	β-1,3X3	15.5 ± 4.32 μM
	β-1,3X4	11.0 ± 3.27 μM

[a] K_d values were determined by ITC in Tris-HCl (10 mM; pH 8.0) containing 100 mM NaCl. The concentration of recombinant proteins was 20 μM. Ligands used were xylose (2 mM), β-1,4X2 (0.2 mM), β-1,4X3 (0.2 mM), β-1,4X4 (0.2 mM), β-1,3X2 (0.8 mM), β-1,3X3 (0.8 mM), and β-1,3X4 (0.8 mM). Experiments were performed in triplicate. ND, not binding detected.

Table S5 Diffraction data and refinement statistics of XylC/β-1,3X2, XylC/β-1,3X3, and XylC/β-1,4X2.

Parameter	Value(s) [a] for:		
	XylC/β-1,3X2	XylC/β-1,3X3	XylC/β-1,4X2
Space group	$P2_12_12_1$	$P2_12_12_1$	$P2_12_12_1$
a, b, c (Å)	61.35,74.38, 85.78	45.61, 84.92, 100.39	45.26, 84.74, 100.12
α, β, γ (°)	90, 90, 90	90, 90, 90	90, 90, 90
Wavelength (Å)	0.9791	0.9791	0.9791
Resolution (Å)	49.90-1.57	37.30-1.36	64.48-1.48
	(1.66-1.57)	(1.40-1.36)	(1.56-1.48)
Redundancy	8.2 (7.5)	10.9 (5.3)	7.8 (6.0)
Completeness (%)	99.9 (100.0)	90.3 (53.3)	97.7 (99.4)
R_{merge} [b]	0.162 (0.908)	0.070 (0.753)	0.088 (0.690)
I/Sigma	9.1 (2.4)	18.9 (1.7)	13.4 (2.7)
R_{work} (%)	0.195 (0.237)	0.216 (0.309)	0.177 (0.223)
R_{free} (%)	0.211 (0.284)	0.234 (0.339)	0.194 (0.254)
Average B-factor	18.52	17.45	18.15
Macromolecules	17.72	16.19	17.49
Solvent	26.73	24.87	26.52
Ligands	12.69	11.22	13.53
Bond lengths (Å)	0.010	0.018	0.007
Bond angles (Å)	1.09	1.37	0.81
Ramachandran Plot			
Favored (%)	98.23	98.22	98.74
Allowed (%)	1.77	1.78	1.26
PDB identifier	8XBB	8XBC	8XBA

[a] Numbers in parentheses refer to data in the highest resolution shell.

[b] $R_{merge} = \Sigma_{hkl}\Sigma_i|I(hkl)_i - \langle I(hkl)\rangle|/\Sigma_{hkl}\Sigma_i \langle I(hkl)_i\rangle$, where I is the observed intensity, $\langle I(hkl)\rangle$ represents the average intensity, and $I(hkl)_i$ represents the observed intensity of each unique reflection.

Table S6 (a separate .xlsx file) Results of DNA motif search from XylRA-containing operons of seven *Vibrio* strains.

Table S7 (a separate .xlsx file) Xyn26A and Xyn26B homologs retrieved from bacterial genomes.

Table S8 (a separate .xlsx file) β-1,3-xylan utilization loci retrieved from bacterial genomes.

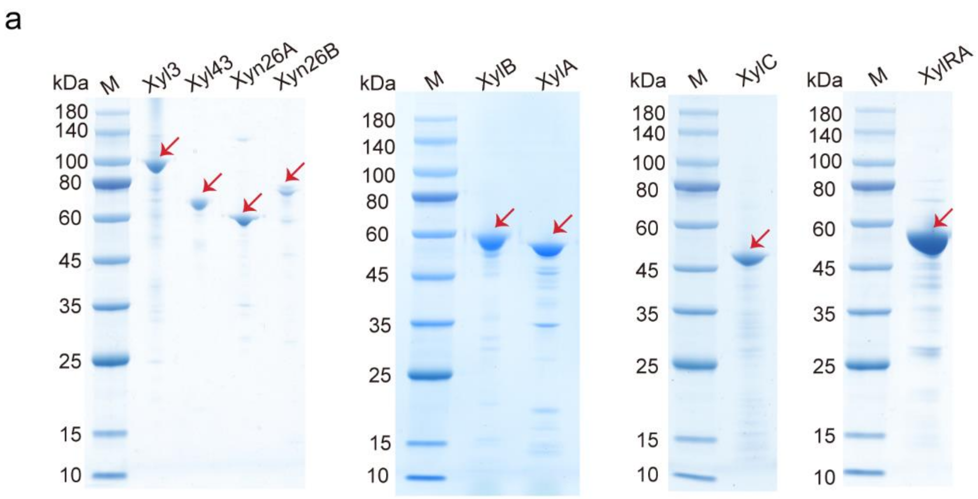

b

Enzyme	Xyn26A	Xyn26A	Xyl43	Xyl3	XylA	XylB
Optimum temperature (°C)	40	40	30	40	50	40
Optimum pH	6.0	6.0	7.0	7.0	7.0	8.0

Fig. S1 SDS-PAGE analysis and biochemical characterization of Xyn26A, Xyn26B, Xyl43, Xyl3, XylA, XylB, XylC, and XylRA. **a** SDS-PAGE analysis. The target proteins were indicated with red arrows. M, protein markers. **b** Biochemical characterization of Xyn26A, Xyn26B, Xyl43, Xyl3, XylA, and XylB. Effect of temperature on the enzyme activity was determined from 10°C to 60°C in PBS (20 mM) under the optimum pH value. Effect of pH was determined in the Britton-Robinson buffer with different pH values (pH 3.0-11.0) at the optimum temperature.

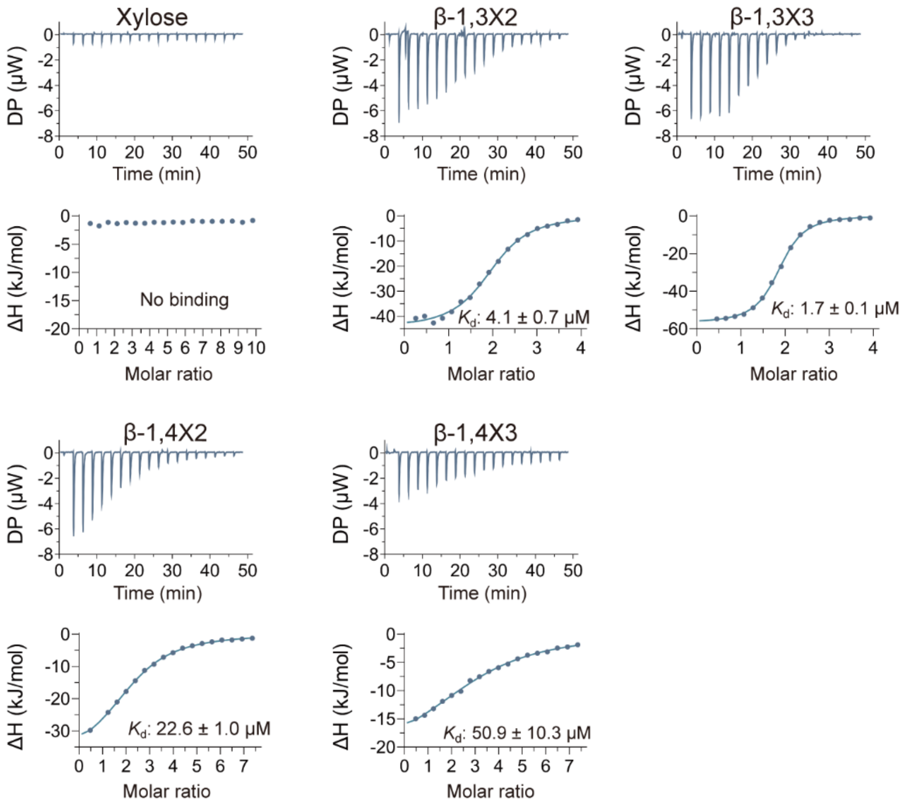

Fig. S2 ITC data for titrations of xylose, β-1,3X2, β-1,3X3, β-1,4X2, and β-1,4X3 into recombinant XylC. ITC traces (top) and integrated binding isotherms (bottom) are shown. The concentration of XylC was 40 μM. The concentrations of xylose, β-1,3X2, β-1,3X4, β-1,4X2, and β-1,4X4 were 2 mM, 0.8 mM, 0.8 mM, 1.5 mM, and 1.5 mM, respectively. Experiments were performed in triplicate and representative results are shown.

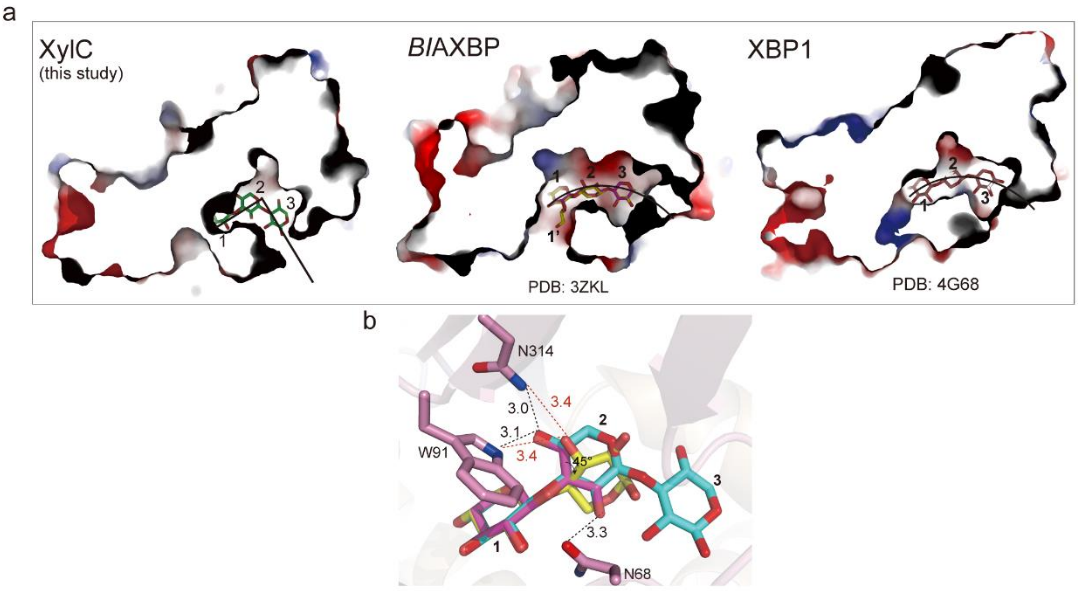

Fig. S3 The substrate binding pocket of XylC. **a** Comparison of substrate binding pockets of XylC, *Bl*AXBP, and XBP1. XylC is specific for β-1,3-xylooligosaccharides and the pocket is L-shaped. Both *Bl*AXBP and XBP1 are specific for β-1,4-xylooligosaccharides and their pockets are arc-shaped. **b** Hydrogen bonding interactions between XylC residues (Asn68, Trp91, and Asn314) and the xylose units 2 of β-1,3X2 (purple), β-1,3X3 (cyan), and β-1,4X2 (yellow). After structural alignment, the xylose unit 2 of β-1,3X2 is overlapped with that of β-1,3X3 and a ~45° deviation of β-1,4X2 was observed. Hydrogen bonds are shown as dashed lines. The ~45° deviation results in the absence or increased distances of hydrogen bonds of β-1,4X2 to Asn68, Trp91, and Asn314.

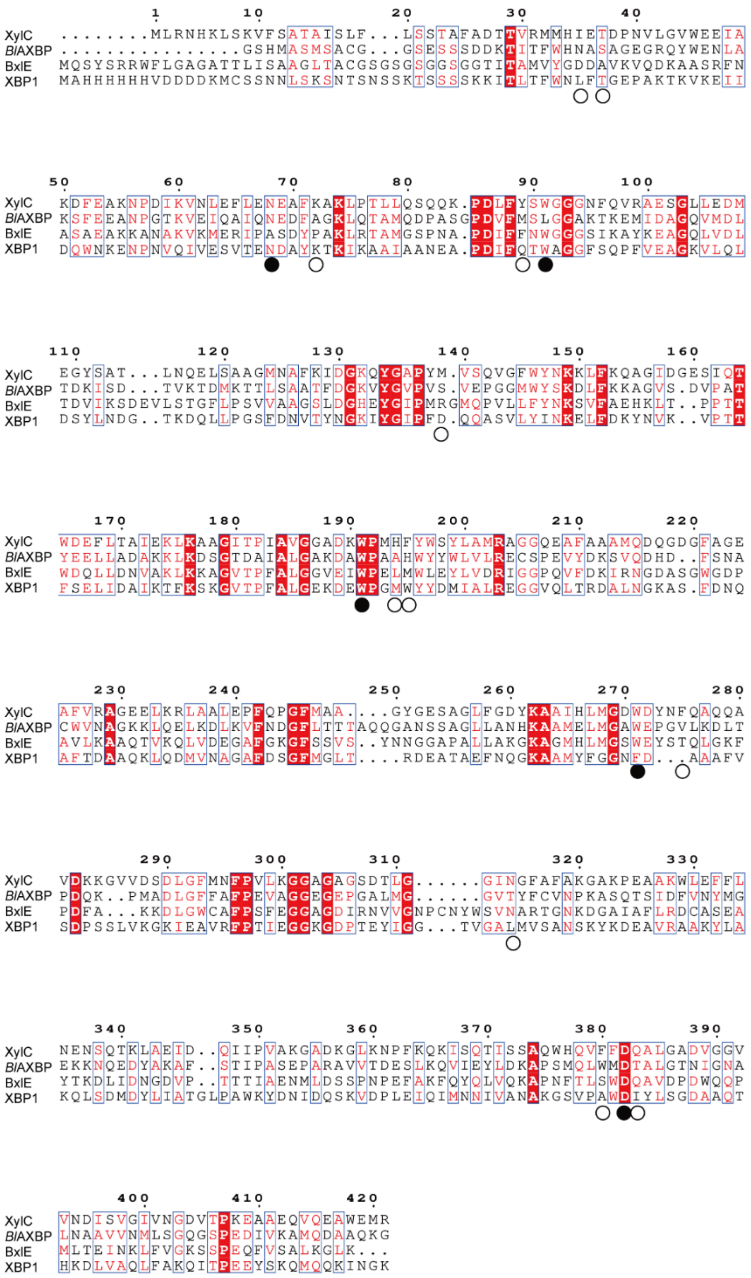

Fig. S4 Multiple sequence alignment of XylC and β-1,4-xylooligosaccharides binding proteins *Bl*AXBP (PDB: 3ZKL), BxlE (PDB: 3VXC), and XBP1 (PDB: 4G68). Key residues of XylC involved in binding β-1,3XOs are indicated with black circles. The solid marks denote relatively conserved residues and the open ones show non-conserved residues.

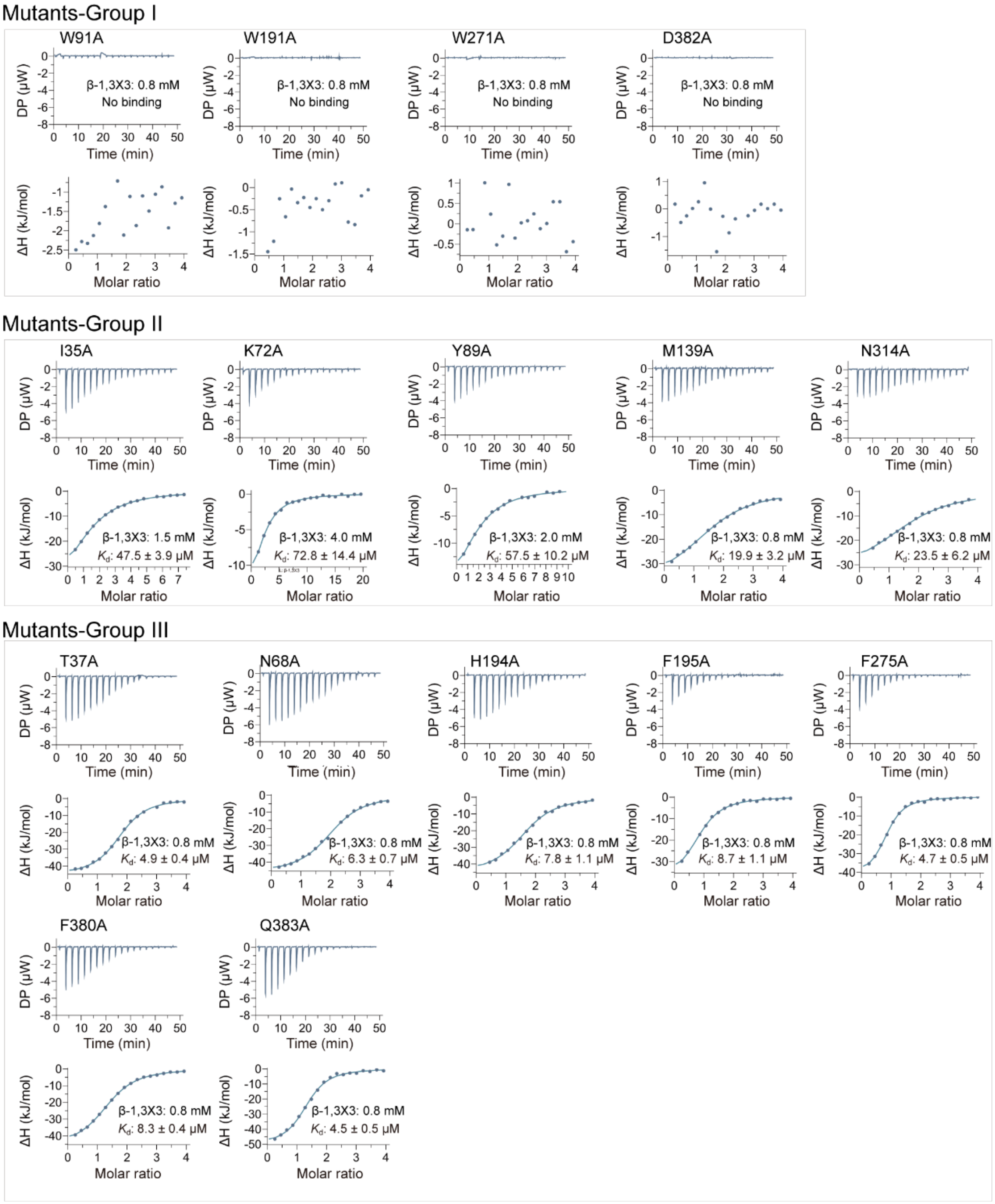

Fig. S5 ITC data for titrations of β-1,3X3 into XylC mutants. ITC traces (top) and integrated binding isotherms (bottom) are shown. The concentration of the mutants was 40 μM. Experiments were performed in triplicate and representative results are shown.

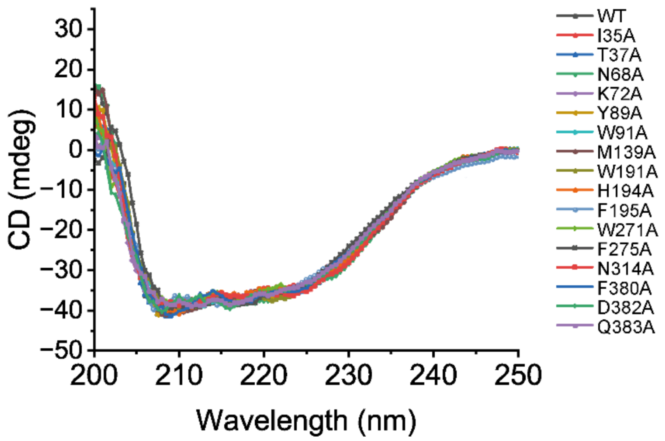

Fig. S6 Circular dichroism (CD) spectra of XylC and its mutants. Experiments were performed in triplicate and representative results are shown.

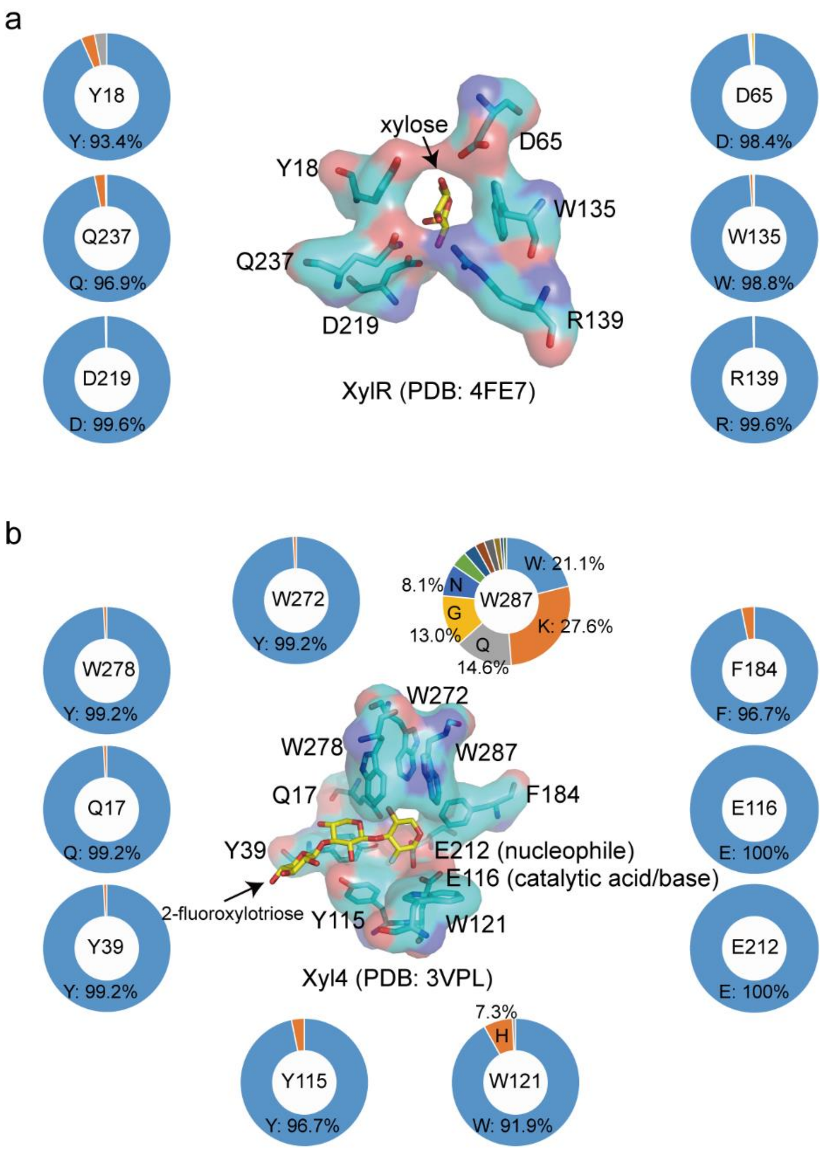

Fig. S7 Analysis of conserved amino acid residues involved in substrate binding and/or catalysis of XylRA-like proteins (**a**) and Xyn26A/Xyn26B-like proteins (**b**). XylRA-like proteins and Xyn26A/Xyn26B-like proteins were analyzed, respectively, according to the structure of XylR (PDB: 4FE7) and Xyl4 (PDB: 3VPL). The pie charts in each panel show the amino acid compositions of corresponding conserved sites in all sequences.

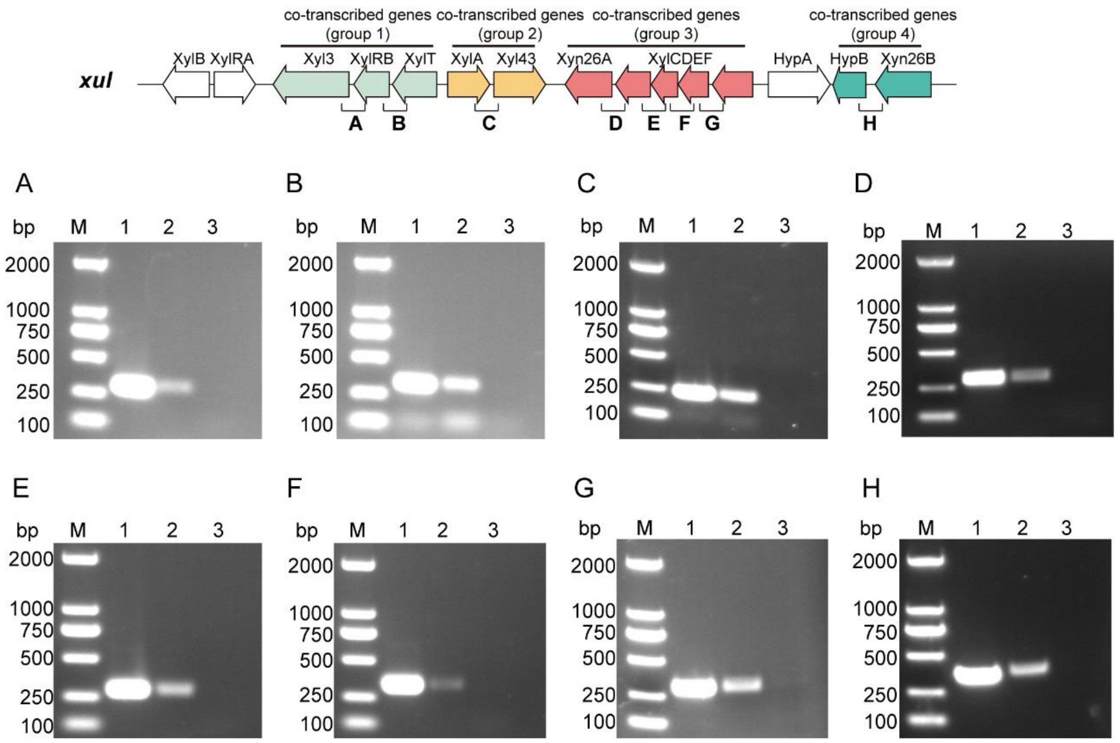

Fig. S8 Analysis of co-transcriptional relationships of *xul* genes in strain EA2. Cells cultivated with xylose as the sole carbon source were used for DNA extraction, RNA extraction and cDNA synthesis. Lanes 1 to 3 correspond to PCR products of intergenic regions with DNA (lane 1, positive control), cDNA (lane 2), and RNA (lane 3, negative control) as the template, respectively. Experiments were performed in triplicate and representative results are shown. The results showed that *xul* contains 4 groups of co-transcribed genes, including 1) *xyl3*, *xylRB*, and *xylT*; 2) *xylA* and *xyl43*; 3) *xyn26A*, *xylF*, *xylE*, *xylD*, and *xylC*; 4) *hypB* and *xyn26B*.

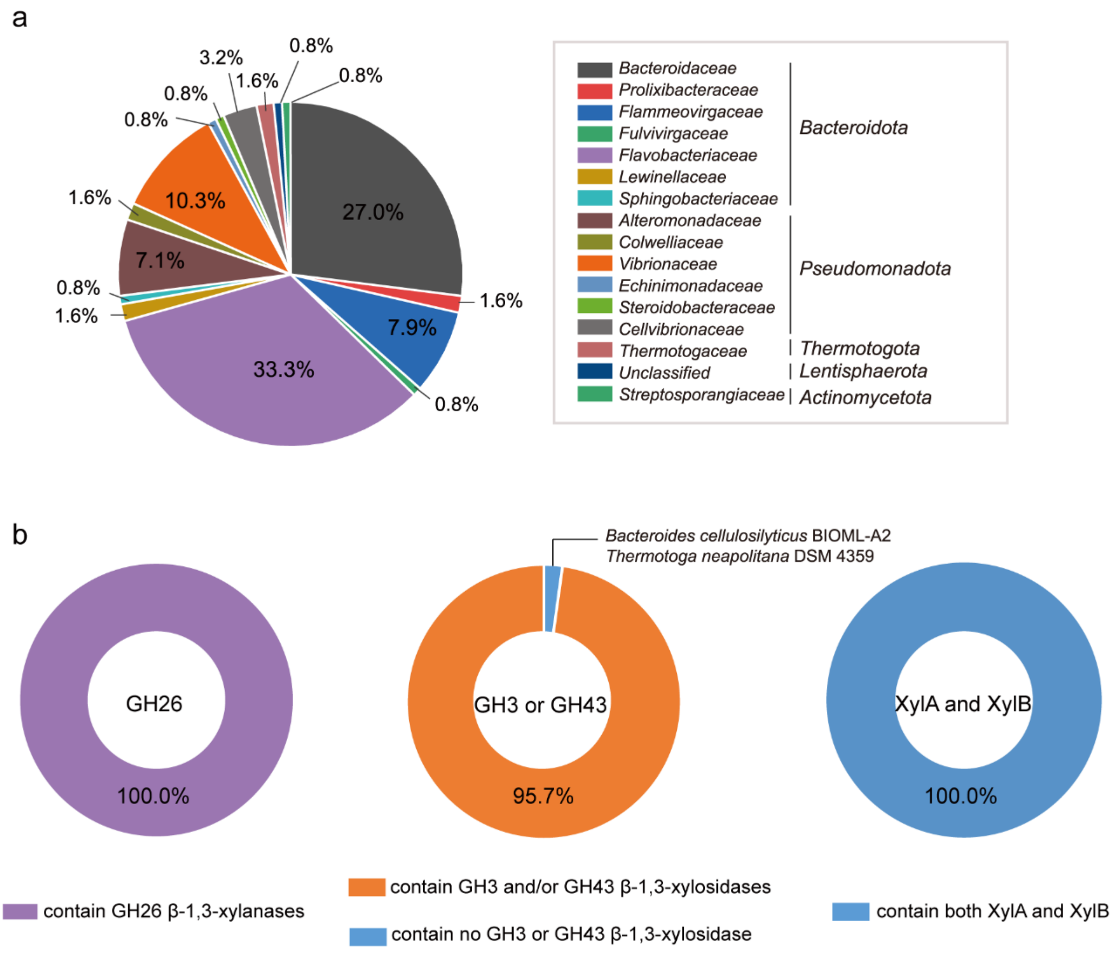

Fig. S9 Statistical analysis of β-1,3-xylanase-containing bacterial genomes (**a**) and key enzymes in β-1,3-xylan utilization loci (**b**). A total of 126 β-1,3-xylanases (Xyn26A/Xyn26B-like sequences) were retrieved from bacterial genomes. The taxonomic affiliations of all bacteria at the family level were analyzed. A total of 47 bacterial genomes containing β-1,3-xylan utilization loci were identified. Key enzymes in these loci involved in β-1,3-xylan utilization, including β-1,3-xylanases (GH26), β-1,3-xylosidases (GH3 or GH43), xylose isomerase (XylA), and xylulokinase (XylB), were analyzed.
